# Iodine-Based Chemical Polymerization Enables the Development of Neat Amorphous Porous Organic Polymers

**DOI:** 10.1021/acsami.4c22197

**Published:** 2025-02-21

**Authors:** Kohei Okubo, Haruka Yoshino, Hitoshi Miyasaka, Hitoshi Kasai, Kouki Oka

**Affiliations:** a Institute of Multidisciplinary Research for Advanced Materials, Tohoku University, 2-1-1 Katahira, Aoba-ku, Sendai, Miyagi 980-8577, Japan; b Institute for Materials Research, Tohoku University, 2-1-1 Katahira, Aoba-ku, Sendai, Miyagi 980-8577, Japan; c Carbon Recycling Energy Research Center, Ibaraki University, 4-12-1 Nakanarusawa, Hitachi, Ibaraki 316-8511, Japan; d Deuterium Science Research Unit, Center for the Promotion of Interdisciplinary Education and Research, Kyoto University, Yoshida, Sakyo-ku, Kyoto 606-8501, Japan

**Keywords:** porous organic polymers, iodine, chemical polymerization, amorphous polymers, open-gate gas adsorption

## Abstract

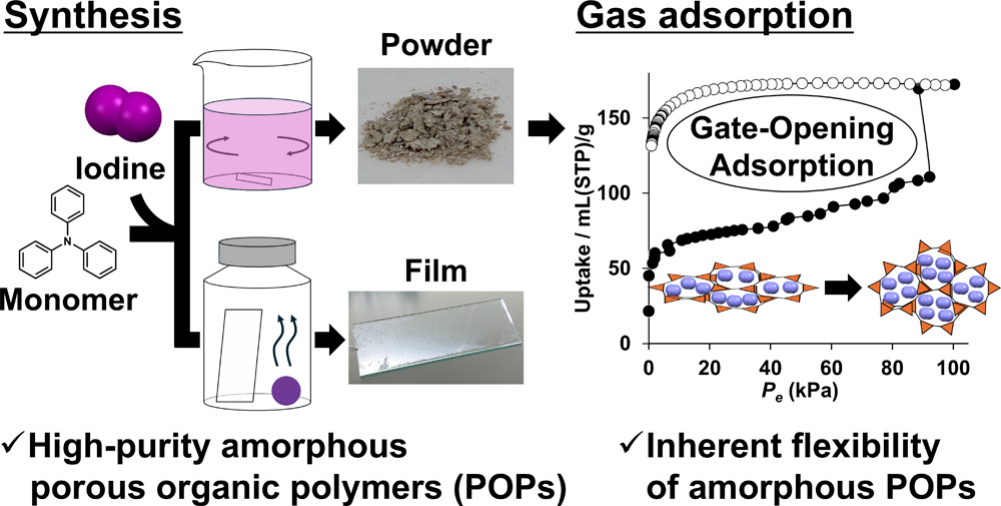

Amorphous porous organic polymers (POPs), with high porosity and high chemical and thermal stability, have been investigated for various applications. Most amorphous POPs are synthesized by electropolymerization or chemical polymerization. However, nonhomogeneous film formation in electropolymerization and residual metal-derived impurities in chemical polymerization are challenges. This study developed a novel chemical polymerization method for amorphous POPs using iodine as an oxidant. Specifically, we synthesized a representative amorphous POP, polytriphenylamine (pTPA). The pTPA was obtained as a powder through solution polymerization and as a thin film via vapor-assisted polymerization. Postreaction, ethanol was used to remove iodine completely. Notably, even though pTPA was constructed from rigid structures, the nitrogen-induced gate-opening phenomenon was exhibited for the first time as amorphous POPs. These results demonstrate that impurity-free amorphous POPs exhibit inherent flexibility against their rigid chemical structure. The novel iodine-based chemical polymerization enables us to synthesize neat amorphous POPs and to explore their pure functions.

## 1 Introduction

Porous organic polymers (POPs),^1,2^ which are porous materials entirely composed of organic molecules, have attracted much attention because they consist only of earth-abundant elements and their pore shapes and functions can be tuned through molecular design.^3^ POPs are categorized based on their crystallinity. Crystalline POPs, also known as covalent organic frameworks (COFs),^4,5^ are constructed via reversible covalent bonding. Based on the crystal structures obtained by X-ray diffraction measurements, many studies have explored the functional development of COFs. Amorphous POPs, constructed via irreversible covalent bonding, exhibit high chemical and thermal stability compared to crystalline POPs and can be classified as hyper-cross-linked polymers (HCPs),^6,7^ porous aromatic frameworks (PAFs),^8,9^ and conjugated microporous polymers (CMPs)^10,11^ based on their structure. These amorphous POPs are primarily synthesized through electropolymerization^12^ (Table 1, top) or chemical oxidative polymerization^13,14^ (Table 1, middle).

**Table 1:** Comparison of Polymerization Methods for Amorphous POPs

**method**	**scalability**	**uniformity**	**purity**
electropolymerization	low	low	high
metal-based chemical oxidative polymerization	high	high	low
iodine-based chemical polymerization (this study)	high	high	high

In electropolymerization (Table 1, top), monomers are electrochemically oxidized and polymerized on electrodes. No oxidants or catalysts are required,^15^ resulting in high-purity polymers. However, maintaining uniform current distribution during polymerization is challenging, and polymerization proceeds locally where the current is concentrated,^16^ making it often difficult to produce homogeneous polymers. On the other hand, in chemical oxidative polymerization (Table 1, middle), oxidation reactions^17^ using iron chloride or aluminum chloride as oxidants have been used for polymerization. Although chemical polymerization has advantages for the large-scale synthesis of homogeneous polymers, the residues of trace amounts of metal components derived from oxidants and catalysts^18,19^ are challenging, and these residues can affect the functionality of amorphous POPs by filling up pores or forming noncovalent bonds with the polymer.^18,20^

In this study, by using iodine as an oxidant, which was easy to remove after polymerization,^21−24^ we established iodine-based chemical polymerization (Table 1, bottom), which retains the advantages of conventional metal-based chemical oxidative polymerization (Table 1, middle) and enabled the production of amorphous POPs with high-purity.

## 2 Experimental Section

### 2.1 Synthesis of Powder **1**

Triphenylamine (248.0 mg, 1.01 mmol) and iodine (771.2 mg, 3.04 mmol) were added to 1,2-dichloroethane (10 mL), and the mixture was stirred for 20 h at 80 °C. The mixture was poured into ethanol. The resulting precipitate was collected by filtration and washed with ethanol to give a light-green powder (49.3 mg). The detailed synthesis procedures of powders **2**–**8** are described in the Supporting Information.

### 2.2 Synthesis of the pTPA Film

Approximately 0.4 mL of a 1,2-dichloroethane solution of 10 mg/mL triphenylamine was spin-coated onto a glass substrate or a glassy carbon substrate at 1500 rpm for 30 s. The TPA-coated substrate was subsequently placed in a preheated chamber with iodine, and it was heated in an oven at a temperature of 80 °C for 1 h. The sample was then washed with ethanol and dried in air at 80 °C.

## 3 Results and Discussion

**Figure 1:**
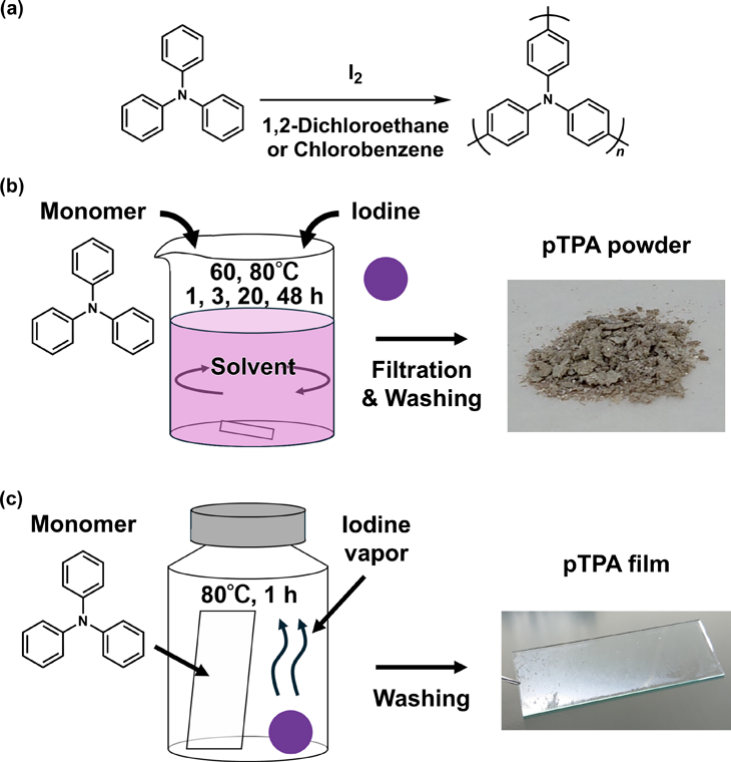
Schematics of (a) the polymerization of p**TPA** and the procedures for (b) solution polymerization and (c) vapor-assisted polymerization.

In this study, chemical polymerization using iodine as an oxidant to synthesize a representative amorphous POP, polytriphenylamine (pTPA), was attempted. pTPA is stable in its one-electron oxidized species, radicals, and is known to exhibit reversible redox activity.^25^ Taking advantage of its high specific surface area, pTPA has been widely investigated for applications in electrocatalysts^26^ and cathode-active materials for lithium-ion batteries.^27^ As shown in Figure S1, the CV of the monomer, TPA, exhibited an onset oxidation potential of 1.15 V vs. SHE, confirming that TPA could be sufficiently polymerized with iodine. As shown in Figure 1, the polymerization of TPA using iodine as an oxidant was carried out by two methods: solution polymerization (Figure 1b) and vapor-assisted polymerization (Figure 1c). First, solution polymerization was conducted to prepare the pTPA powder. TPA was polymerized in 1,2-dichloroethane or chlorobenzene, which are widely used as solvents in chemical polymerization,^26^ and the reaction parameters such as temperature, time, and iodine amount were varied (Table 2). Following polymerization, the precipitated pTPA was collected via filtration and washed with ethanol to obtain the pTPA powder (powders **1**–**8**) as a green or yellow powder.

**Table 2:** Synthesis of **p**TPA**** Powders under Different Reaction Conditions^a^

sample	solvent	amount of iodine (mmol)	temperature (°C)	time (h)	BET surface area (m^2^ g^–1^)
powder 1	1,2-dichloroethane	3	80	20	41.842
powder 2	1,2-dichloroethane	5	80	20	(2.7 ± 0.2) × 10^2^
powder 3	1,2-dichloroethane	20	80	20	204.74
powder 4	1,2-dichloroethane	5	80	1	47.568
powder 5	1,2-dichloroethane	5	80	3	98.462
powder 6	1,2-dichloroethane	5	80	48	252.80
powder 7	1,2-dichloroethane	5	60	20	32.233
powder 8	chlorobenzene	5	80	20	11.575

a Reaction condition: triphenylamine (1 mmol) and iodine in each solvent (10 mL).

**Figure 2:**
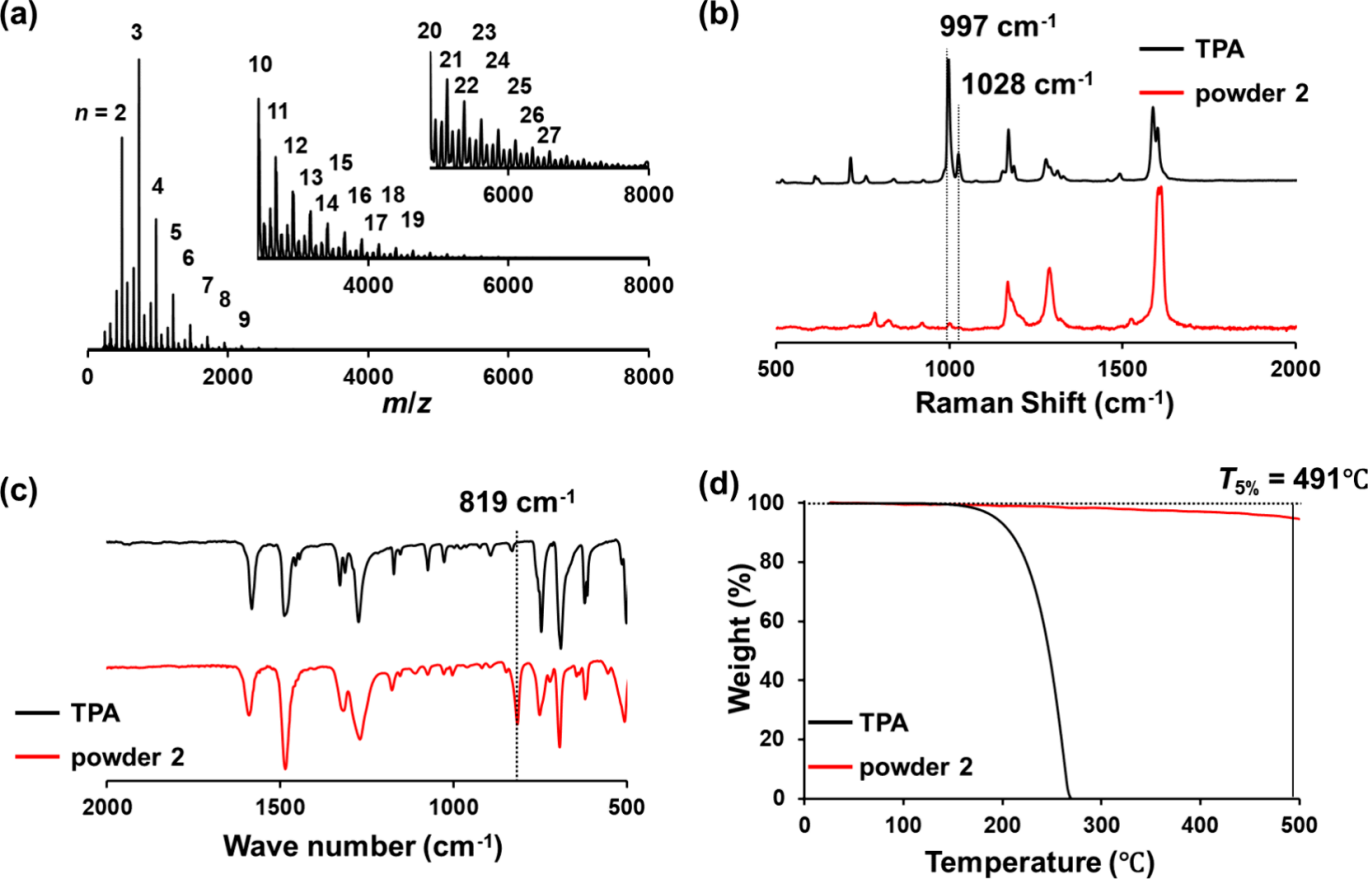
(a) MALDI-TOF MS spectrum of powder **2**. *n* refers to the number of triphenylamine units in the polymer; therefore, the difference between the main segment peaks is 245.3. The peaks among the main segment peaks indicate fragmentation between C–N bonds.^33^ (b) Raman spectra, (c) IR spectra, and (d) TG curves of TPA (black) and powder **2** (red).

The progress of polymerization was confirmed by matrix-assisted laser desorption ionization-time-of-flight mass (MALDI-TOF MS) spectrometry, Raman spectroscopy, infrared (IR) spectroscopy, and solid-state ^13^C NMR. As a representative example, results for powder **2** are summarized in Figure 2a–c. First, as shown in Figure 2a, the MALDI-TOF MS spectrum indicated the formation of polymers consisting of at least 27 TPA units, exhibiting a higher degree of polymerization than previous pTPA synthesized by conventional oxidative polymerization.^28^ In Figure 2b, the Raman spectra demonstrated that the peaks for the C–H bending vibration (997 cm^–1^) and ring deformation vibration (1028 cm^–1^),^29^ which are associated with monosubstituted benzene derived from the monomer, decreased, indicating that the monosubstituted benzene on TPA was reduced during the reaction. Subsequently, as shown in Figure 2c, the IR spectra demonstrated a decrease in the peak for the C–H bending vibration of monosubstituted benzene (747 cm^–1^) derived from TPA and the appearance of a new peak for the C–H bending vibration of the 1,4-disubstituted benzene (819 cm^–1^),^29^ confirming that the monosubstituted benzene decreased and 1,4-disubstituted benzene formed on TPA. The changes in these spectra (Figure 2b,c) indicate that polymerization proceeded at the para position of the benzene ring in TPA. In Figure S2, the solid-state ^13^C NMR spectrum showed three peaks at 126.45, 136.02, and 147.11 ppm, corresponding to aromatic carbons, indicating that there was no significant difference in the molecular structure from the previously reported pTPA.^30^

As shown in Figure S3, the reaction progress for each polymerization condition was semiquantified based on the percentage decrease in absorbance in the IR spectra of monosubstituted benzene at the C–H bending vibration (747 cm^–1^). In Table S2, the comparison of powders **1**–**3** indicates that the reaction proceeded more rapidly as the iodine amount increased, whereas the reaction slowed down when the iodine amount exceeded 5 equiv per 1 molecule of TPA. Comparisons of powder **2** with powders **4** and **5** suggest that polymerization occurred primarily within the first hour with the reaction gradually continuing thereafter. Comparisons of powder **2** with powder **6** indicate that polymerization does not proceed further with reaction time extended beyond 20 h. The comparison of powders **2** and **7** shows that higher reaction temperatures promoted the reaction, with the boiling point of 1,2-dichloroethane (84 °C) limiting experiments at temperatures above 80 °C. The comparison of powders **2** and **8** reveals that the synthesis progressed more efficiently in 1,2-dichloroethane than in chlorobenzene. This result was presumably attributed to the higher solubility of both monomers and polymers in 1,2-dichloroethane.

MALDI-TOF MS measurements using a fixed laser power and the degree of polymerization were compared across powders **1**–**8**. As shown in Figures S4 and S6, powders **1** and **4** exhibited dimer peaks as the main peaks, and polymers consisting of a maximum of 12 TPA units were formed, indicating a relatively low degree of polymerization. In the region above 2000 molecular weight, as shown in Figure 2a and Figures S5, S8, and S9, powders **2**, **3**, **6**, and **7** formed polymers consisting of more than 20 TPA units. The above tendency of the degree of polymerization was consistent with the reaction progress (Table S2) semiquantified from the IR spectra. The thermal stability of the polymers was evaluated using TG analysis under a nitrogen atmosphere, and the result for powder **2** is shown in Figure 2d as a representative example. The powder **2** had a 5% weight loss temperature (*T*_5%_) of 491 °C, supporting polymer formation and exhibiting similar thermal stability as the pTPA in previous studies.^17,26,28^

Furthermore, as shown in Figure S11, the SEM-EDX spectrum revealed no peaks attributed to impurities, such as metals or iodine, including the iodine L line, indicating that the residual impurities in powder **2** were below 0.1 atm%, the detection limit. Similar low impurity levels were confirmed for the other powders. Additionally, in Figure S12, the Raman spectrum demonstrated no peaks corresponding to iodine (100–200 cm^–1^^31^), and in Figure S13, the XPS spectrum showed no peaks corresponding to the iodine 3d line (618.8 and 623.5 eV^32^), further supporting the absence of residual impurities. As shown in Figure S14, no obvious peaks were observed in the powder X-ray diffraction (XRD) pattern of powder **2**, indicating that powder **2**, as with other pTPA synthesized by other polymerization methods,^17^ consisted of an amorphous structure, resulting from irreversible bonding. As shown in Figure S15, the SEM images of powder **2** revealed a rough surface structure of powder **2**. As shown in Figure S15b, the microscopic voids existing on the surface of powder **2** support the high BET specific surface area of powder **2** (also see the gas adsorption measurement described below).

Next, vapor-assisted polymerization was applied to prepare pTPA films. Conventionally, in chemical polymerization, thin films are prepared by coating an oxidant on a substrate in advance and exposing it to monomer vapor.^34^ However, due to the high boiling point of TPA (347 °C), which makes it difficult to evaporate, it has been challenging to prepare a pTPA thin film by chemical polymerization. Therefore, this study focused on the development of a method for preparing thin films of amorphous POPs containing pTPA, and the reaction condition was based on those for solution polymerization. TPA in 1,2-dichloroethane solution was spin-coated onto glass or glassy carbon substrates, followed by exposure to iodine vapor at 80 °C for 1 h in a sealed container (Figure 1c). Postpolymerization, the pTPA film exhibited a dark red color due to oxidation, which was reduced and turned colorless by annealing at 80 °C and washing with ethanol to remove excess oxidant. This study developed a vapor-assisted polymerization method using sublimable iodine as an oxidant, enabling the successful preparation of pTPA thin films via chemical polymerization for the first time.

The progress of polymerization was confirmed by MALDI-TOF MS measurements. As shown in Figure S16, the MALDI-TOF MS spectrum indicates the formation of polymers consisting of at least six TPA units. Additionally, Figure S17 shows no peaks in the XRD pattern of the obtained pTPA film, indicating that it consists of an amorphous structure, similar to the pTPA powder. As shown in Figure S18, the SEM image of the pTPA film revealed a smooth surface structure of pTPA.

**Figure 3:**
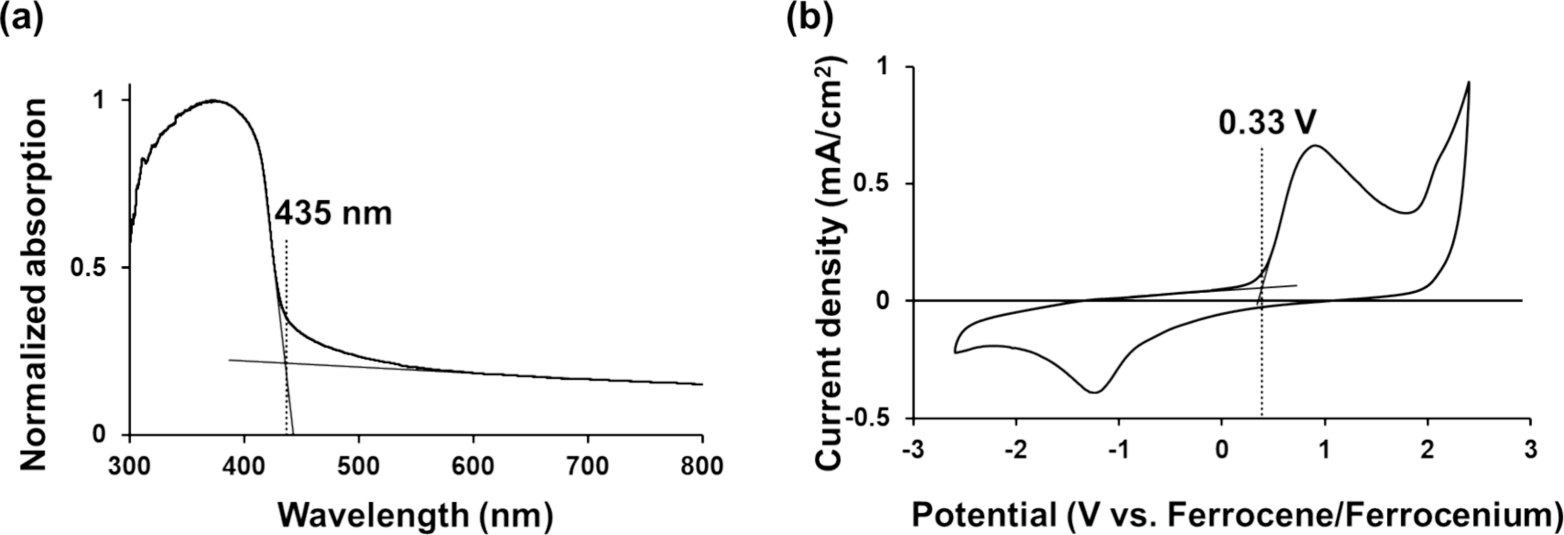
(a) UV–vis spectrum and (b) cyclic voltammogram of the pTPA film. The CV was recorded at a scan rate of 50 mV/s in acetonitrile (0.1 M TBAPF_6_).

To investigate the electronic structure of the pTPA, UV–vis spectroscopy and CV measurements were performed (Figure 3). As shown in Figure 3a, the UV–vis spectrum indicates that the pTPA film has an absorption edge at 435 nm and a band gap of 2.85 eV. Furthermore, as shown in Figure 3b, the cyclic voltammogram demonstrates that the onset oxidation potential of the pTPA film was 1.03 V vs. SHE. These results indicate that the highest occupied molecular orbital (HOMO) and lowest unoccupied molecular orbital (LUMO) levels of the pTPA film were −5.04 and −2.19 eV, respectively. Compared to previously reported data^35^ obtained from measurements in solution, the band gap decreased from 3.08 to 2.85 eV, the HOMO level deepened from −4.86 to −5.04 eV, and the LUMO level deepened from −1.78 to −2.18 eV. This is presumably ascribed to the large overlap of π orbitals in the aggregated state.^36^ In this study, the fabrication of pTPA films enabled precise clarification of the band structure in the solid state. In our continuing study, we explore the synthesis of pTPA films under varying conditions to further tune their electronic structure properties.

**Figure 4:**
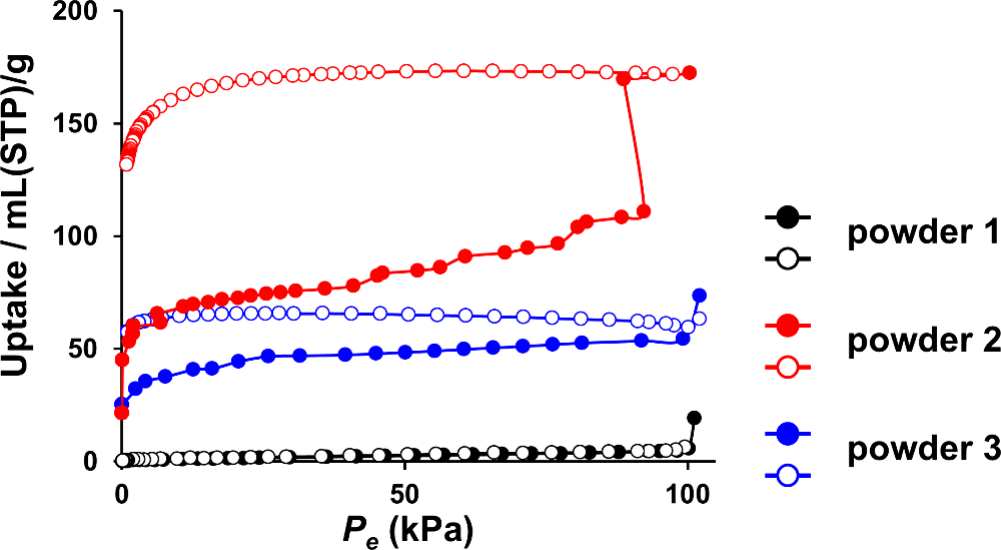
N_2_ adsorption isotherms of powder **1** (black), powder **2** (red), and powder **3** (blue), measured at 77 K. Solid symbols denote the adsorption isotherms, and open symbols denote the desorption isotherms. As shown in Figure S21, even when N_2_ adsorption measurements were repeated, the gate-opening gas adsorption behavior was reproduced, which confirmed the reliability of the measurements and supported a reversible structural transition.

As shown in Figure 4 and Figure S19, N_2_ adsorption measurements at 77 K on powders **1**–**8** were used to calculate the BET specific surface area. As listed in Table 2, the BET surface area increases with the degree of polymerization progress, as indicated in Table S2, with a maximum in powder **2** (Figure 4, red). This result is higher than or comparable to other pTPA samples^26,35^ synthesized by conventional chemical polymerization using iron chloride as an oxidant. The suggestion that the complete removal of impurities likely contributed to the high porosity. In powder **3**, where polymerization had progressed further, the smaller BET surface area could be attributed to a reduction in pore size due to excessive polymerization, resulting in inaccessible voids for the adsorbate.^37^ Notably, powder **2**, at a relative pressure of 0.9, exhibited gate-opening gas adsorption behavior,^38^ with a steep increase in the adsorption volume. This change in adsorption corresponds to an increase in the number of nitrogen molecules adsorbed per TPA unit from 1.20 to 1.86 molecules (Figure S20). The gate-opening phenomenon is a transition from a closed-porous structure to an open-porous structure at a specific pressure, leading to a significant increase in adsorption.^39,40^ This is the first time that this phenomenon has been observed in amorphous POPs. Previously, porous materials, exhibiting the nitrogen-induced gate-opening phenomenon, have been limited to crystalline materials such as metal–organic frameworks (MOFs)^38^ and COFs. The N_2_ molecules have no strong interaction with the framework, such as dipole or quadrupole moments,^41^ and therefore, the gate-opening phenomenon by nitrogen adsorption requires exceptionally high degrees of flexibility for the chemical structure in porous materials. In MOFs, their coordination bonds are usually weak, allowing the construction of highly flexible structures, but their structures are usually unstable and often collapse through repeated adsorption and desorption.^42^ On the other hand, the covalent bonds in COFs are strong and rigid, and therefore, constructing a stable structure is easy, but constructing a flexible structure is difficult.^43^ Therefore, COFs exhibiting nitrogen-induced gate-opening adsorption behavior have been limited to those that contain flexible bonds such as C=N double bonds^44^ and C–O single bonds.^40^ In contrast, the pTPA obtained in this study showed a gate-opening phenomenon triggered by nitrogen adsorption, even though they were constructed from rigid structures consisting of directly bonded benzene rings of rigid triphenylamine.^45^ The results of this study indicate that metal-derived impurities inhibited the dynamic structural changes of amorphous POPs, and the inherent flexibility of amorphous materials can be exhibited by removing these impurities, which led to the emergence of the nitrogen-induced gate-opening phenomenon. Therefore, the results are the first demonstration of the structural flexibility of POPs due to their amorphous structure by gas absorption, which is a novel finding in gas adsorption and opens a new research field for the functional development of amorphous porous materials. In powder **2**, the BET specific surface area was calculated based on the adsorption isotherm of a closed-porous structure, and therefore, the BET specific surface area of powder **2** was likely to be underestimated.^35^

**Figure 5:**
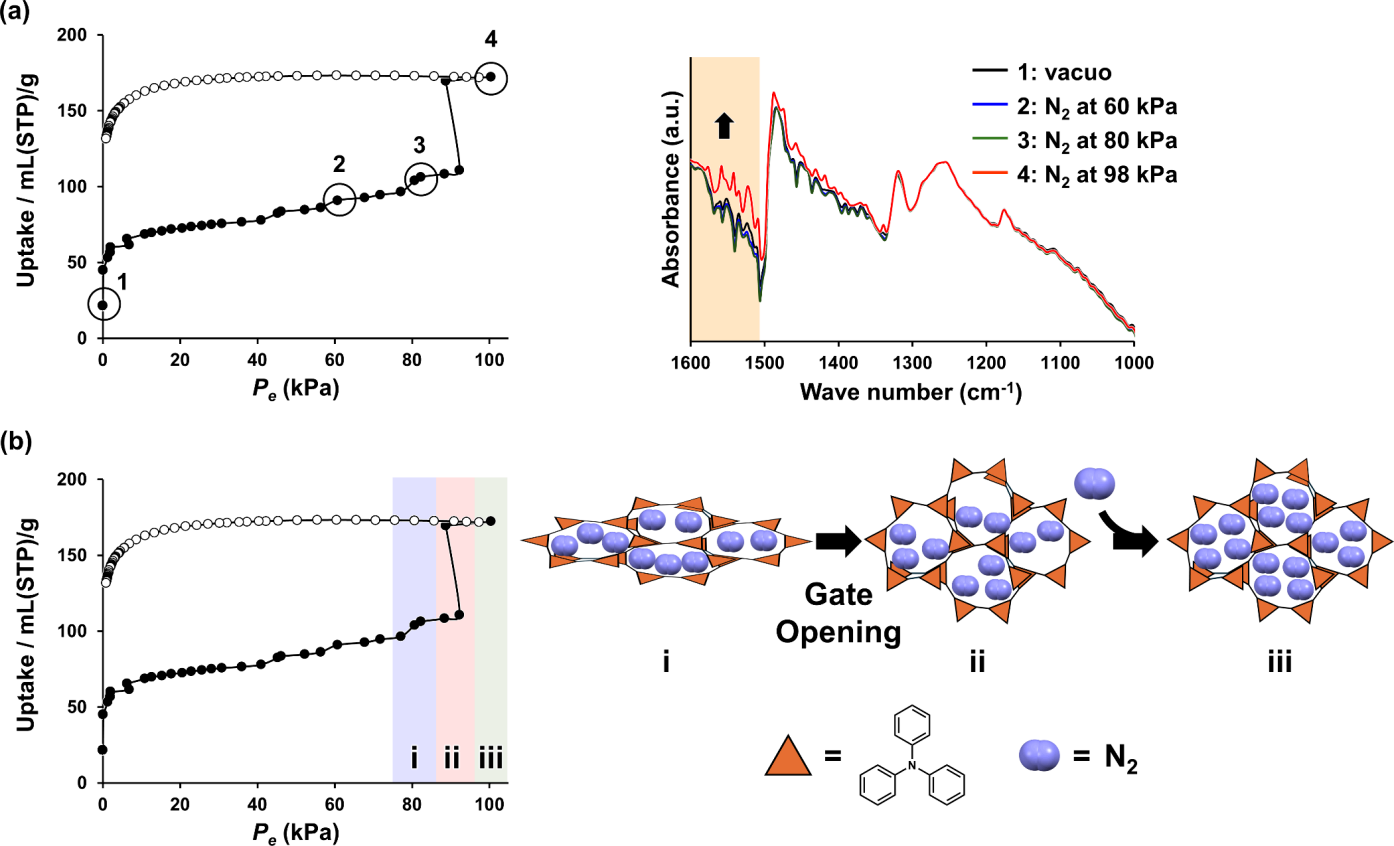
(a) IR spectra recorded for powder **2** under vacuum (black), 60 kPa (blue), 80 kPa (green), and 98 kPa (red) N_2_ atmospheres at 77 K. The orange area represents the range of wavenumbers with increased peak intensity. (b) Schematic images of the structural transition for pTPA induced by the nitrogen adsorption.

In Figure 5a, the *in situ* IR measurement of powder **2** under a nitrogen atmosphere demonstrated no change in the IR spectrum from vacuum to an 80 kPa nitrogen atmosphere. However, at a 98 kPa nitrogen atmosphere, the peak intensity in the 1500–1600 cm^–1^ region, corresponding to the C–H stretching and C–H bending vibrations of the benzene ring (Figure 5a, orange), increased, which triggered the gate-opening phenomenon. This result suggests a decrease in the C–H···π interactions between the benzene rings in the triphenylamine backbone. Similar IR spectral changes have been observed in the structural changes of COFs that show pressure-dependent changes in luminescence.^46^ As shown in Figure S22, the substructure of pTPA, optimized by density functional theory (DFT) calculations using the B3LYP functional and the 6-31G(d,p) basis set, supports that the pTPA network expands in a three-dimensional direction rather than two dimensions due to the torsion between the triphenylamine structures. Porous structures with three-dimensional frameworks are generally known to be less structurally stable than two-dimensional structures and are prone to pore shrinkage.^47^ In the pTPA synthesized in this study, pore shrinkage occurred similarly, and the pores are considered to have recovered as a result of gas molecule adsorption (Figure 5b).

## 4 Conclusions

In this study, novel chemical polymerization using iodine as an oxidant was developed and pTPA, a representative amorphous POP, was successfully synthesized. The iodine used in the polymerization was facilely removed by simple immersion in a solvent after the synthesis, resulting in pure pTPA with no residual impurities. The iodine-based chemical polymerization method has the potential to be applied to a wide range of monomers, which have been utilized in conventional metal-based chemical oxidative polymerization methods. For example, as shown in Figure S23, this polymerization method has been confirmed to be employed in the synthesis of other amorphous POPs (e.g., polythiophenes and polytriphenylamines), which indicates its broad applicability. Notably, pTPA, synthesized in this study, exhibited gate-opening N_2_ adsorption behavior, marking the first time that this phenomenon has been observed in amorphous POPs. This result supports the high stimulus responsiveness of the obtained amorphous POPs and indicates that they are promising for applications to stimulus-responsive materials, such as highly sensitive sensing materials taking advantage of changes in fluorescence based on dynamic structural transformation,^48^ and separation materials for specific gases through gate-opening adsorption.^49^ Furthermore, the high chemical and thermal stability of amorphous POPs derived from irreversible covalent bonds enables amorphous POPs to construct stimulus-responsive materials with high durability compared to MOFs and COFs, which are constructed from reversible bonds. The novel iodine-based chemical polymerization enables the synthesis of neat amorphous POPs and the exploration of their pure functions.

## Supporting Information Available

Detailed synthesis procedures of powders **2**–**8**, cyclic voltammogram, solid-state ^13^C NMR spectrum, IR absorbance spectra, MALDI spectra, SEM-EDX spectra, Raman spectra, XPS spectra, XRD patterns, SEM images, and N_2_ adsorption isotherms (DOCX)
