# Supplementary material for: Iodine-Based Chemical Polymerization Enables the Development of Neat Amorphous Porous Organic Polymers
Source: ACS Appl Mater Interfaces. 2025 Feb 21;17(9):14561–8. doi: 10.1021/acsami.4c22197 (PMC11891841; doi:10.1021/acsami.4c22197)
Supplement: Supplementary file 1 — am4c22197_si_001.docx [file am4c22197_si_001.docx]

Supporting Information

**Iodine-Based Chemical Polymerization Enables the Development of Neat Amorphous Porous Organic Polymers**

Kohei Okubo,^[a]^ Haruka Yoshino,^[b]^ Hitoshi Miyasaka,^[b]^ Hitoshi Kasai,^[a]^ and Kouki Oka*^[a, c, d]^

[a] Institute of Multidisciplinary Research for Advanced Materials,

Tohoku University

2-1-1 Katahira, Aoba-ku, Sendai, Miyagi 980-8577, Japan

E-mail: oka@tohoku.ac.jp (Kouki Oka)

[b] Institute for Materials Research,

Tohoku University

2-1-1 Katahira, Aoba-ku, Sendai, Miyagi 980-8577, Japan

[c] Carbon Recycling Energy Research Center,

Ibaraki University

4-12-1 Nakanarusawa, Hitachi, Ibaraki 316-8511, Japan

[d] Deuterium Science Research Unit, Center for the Promotion of Interdiscipli-nary Education and Research,

Kyoto University

Yoshida, Sakyo-ku, Kyoto 606-8501, Japan

1. **Measurements.**

Cyclic voltammogram (**CV**) was recorded with a three-electrode system using glassy carbon disk electrode as the working electrodes, a Pt wire as the counter electrode, an Ag/Ag^+^ as the reference electrode, and acetonitrile solvent containing TBAPF_6_ was used as the electrolyte (0.1 M).

Matrix-assisted laser desorption ionization-time of flight mass (**MALDI-TOF MS**) was performed on Shimadzu MALDI-8030.

Infrared (**IR**) spectra were recorded on Shimadzu IRSpirit equipped with an ATR accessory.

Raman spectra were recorded on Renishaw inVia confocal Raman microscope system with lasers emitting at 532 nm.

Thermogravimetric (**TG**) analyses were performed with a Shimadzu DTG-60A at a heating rate of 1 ℃/min under nitrogen.

Scanning electron microscopy (**SEM**) images (accelerating voltage of 1 kV) and energy-dispersive X-ray (**EDX**) spectra (accelerating voltage of 15 kV) were obtained using the HITACHI SU6600.

X-ray diffraction (**XRD**) analyses were performed with a Rigaku SmartLab X-ray diffraction system. UV-vis spectra were recorded on Shimadzu UV-1900i.

Gas adsorption measurements were performed on a MicrotracBEL BELSORP-max X. The adsorption isotherm for N_2_ was collected at 77 K. Before all measurements, the samples were dried under reduced pressure and 353 K for 3 h. To calculate the standard deviation, nitrogen adsorption measurements were carried out four times, and the Brunauer-Emmett-Teller (**BET**) specific surface area was calculated for each. As a representative example, in the measurement of four samples of **powder 2**, the **BET** specific surface areas were 273.39, 245.51, 298.41, and 252.51 m^2^/g, and the average of the four measurements was 267 m^2^/g, with a standard deviation of 21 m^2^/g. Therefore, the **BET** specific surface area of **powder 2** was (2.7±0.2) ×10^2^ m^2^/g.

*In situ* **IR** spectra were obtained using CaF_2_ windows on a cryostat system (RC102, CRYO Industries) connected to a gas handling and pressure monitoring system (BELSORP MAX; MicrotracBEL) with a transmission configuration using a JASCO FT-IR 4200 spectrometer. The sample was sandwiched between two CaF_2_ plates.

1. **Materials.**

Triphenylamine, iodine, chlorobenzene, and tetrabutylammonium hexafluorophosphate (**TBAPF_6_**) were purchased from Tokyo Chemical Industry. 1,2-Dichloroethane, ethanol, and acetonitrile were purchased from Nacalai tesque.

1. **Synthesis of powder 2**

Triphenylamine (252.3 mg, 1.03 mmol) and iodine (1310 mg, 5.16 mmol) were added to 1,2-dichloroethane (10 mL) and the mixture was stirred for 20 h at 80 °C. The mixture was poured into ethanol. The resulting precipitate was collected by filtration and washed with ethanol, to give a light-yellow powder (93.4 mg).

1. **Synthesis of powder 3**

Triphenylamine (257.1 mg, 1.05 mmol) and iodine (5160 mg, 20.3 mmol) were added to 1,2-dichloroethane (10 mL) and the mixture was stirred for 20 h at 80 °C. The mixture was poured into ethanol. The resulting precipitate was collected by filtration and washed with ethanol, to give a light-brown powder (153.2 mg).

1. **Synthesis of powder4**

Triphenylamine (254.0 mg, 1.04 mmol) and iodine (1279 mg, 5.04 mmol) were added to 1,2-dichloroethane (10 mL) and the mixture was stirred for 1 h at 80 °C. The mixture was poured into ethanol. The resulting precipitate was collected by filtration and washed with ethanol, to give a light-green powder (28.61 mg).

1. **Synthesis of powder 5**

Triphenylamine (260.1 mg, 1.06 mmol) and iodine (1271 mg, 5.01 mmol) were added to 1,2-dichloroethane (10 mL) and the mixture was stirred for 3 h at 80 °C. The mixture was poured into ethanol. The resulting precipitate was collected by filtration and washed with ethanol, to give a light-green powder (36.72 mg).

1. **Synthesis of powder 6**

Triphenylamine (249.9 mg, 1.02 mmol) and iodine (1285 mg, 5.06 mmol) were added to 1,2-dichloroethane (10 mL) and the mixture was stirred for 48 h at 80 °C. The mixture was poured into ethanol. The resulting precipitate was collected by filtration and washed with ethanol, to give a light-green powder (121.6 mg).

1. **Synthesis of powder 7**

Triphenylamine (254.9 mg, 1.04 mmol) and iodine (1295 mg, 5.10 mmol) were added to 1,2-dichloroethane (10 mL) and the mixture was stirred for 20 h at 60 °C. The mixture was poured into ethanol. The resulting precipitate was collected by filtration and washed with ethanol, to give a light-yellow powder (128.3 mg).

1. **Synthesis of powder 8**

Triphenylamine (248.5 mg, 1.01 mmol) and iodine (1323 mg, 5.21 mmol) were added to chlorobenzene (10 mL) and the mixture was stirred for 20 h at 80 °C. The mixture was poured into ethanol. The resulting precipitate was collected by filtration and washed with ethanol, to give a light-green powder (124.2 mg).

1. **Synthesis of pTPA film**

Approximately 0.4 mL of a 1,2-dichloroethane solution of 10 mg/mL triphenylamine was spin coated onto a glass substrate or a glassy carbon substrate at 1500 rpm for 30 s. The **TPA**-coated substrate was subsequently placed in a pre-heated chamber with iodine, and it was heated in an oven at a temperature of 80 °C for 1 h. The sample was then washed with ethanol and dried in air at 80°C.


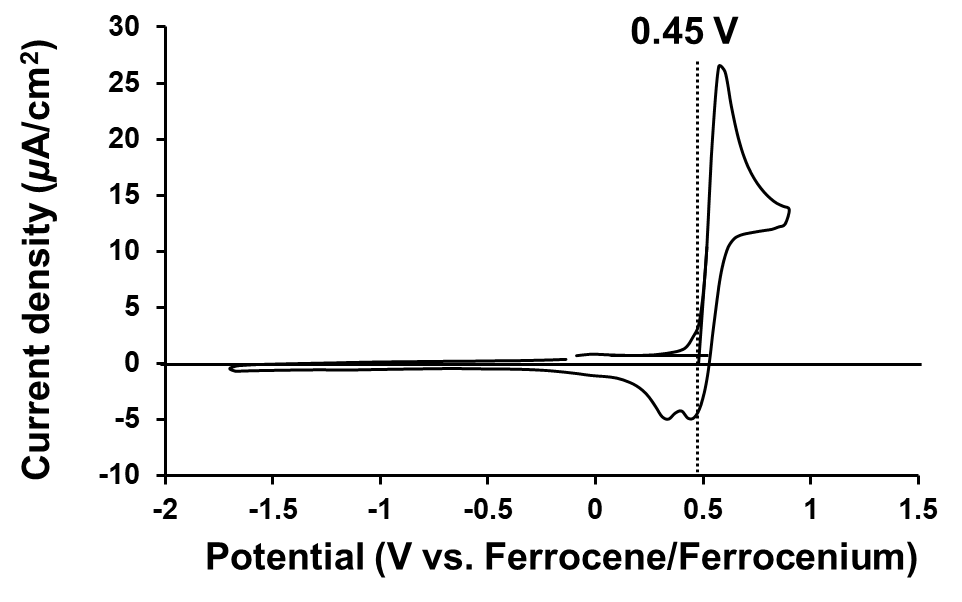


**Figure S1.** Cyclic voltammogram of **TPA** in acetonitrile (0.1 M **TBAPF_6_**). Scan rate 50 mV/s.


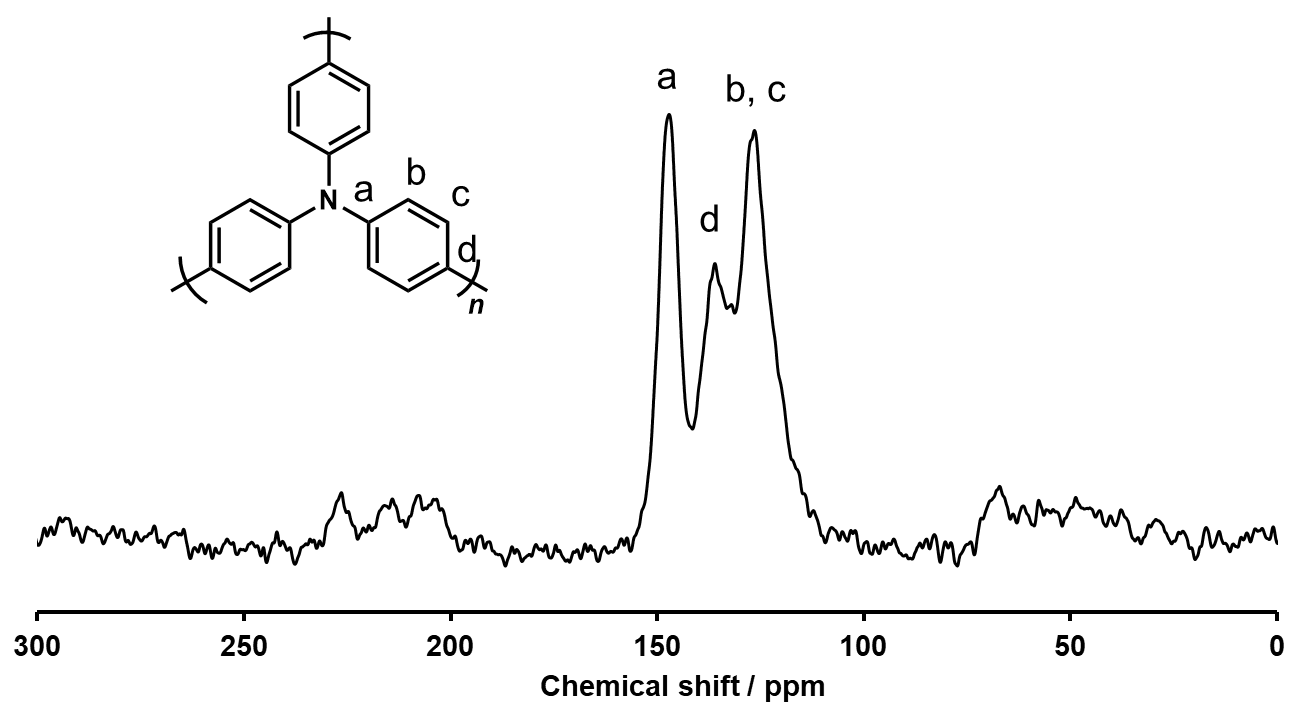


**Figure S2.** Solid-state ^13^C **NMR** spectrum of **powder 2**. Broad peaks around 60 and 210 ppm indicate spinning sidebands.


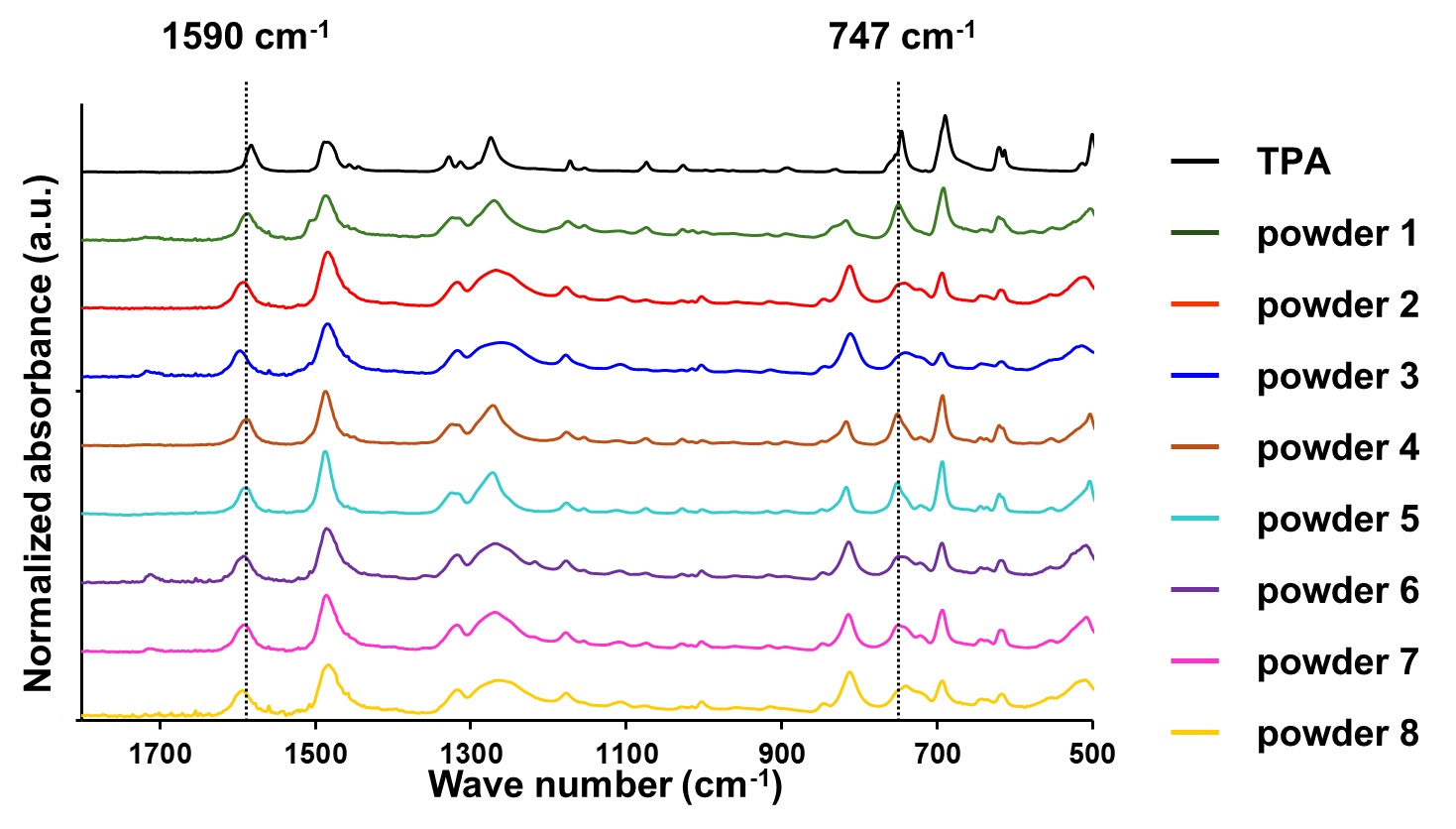


**Figure S3. IR** absorbance spectra of **TPA** and **powders 1–8**, normalized by the peaks around 1590cm^-1^. The peaks at 747 cm^-1^ and 1590 cm^-1^ correspond to the C-H bending vibration of monosubstituted benzene and the C=C stretching vibration of benzene, respectively.


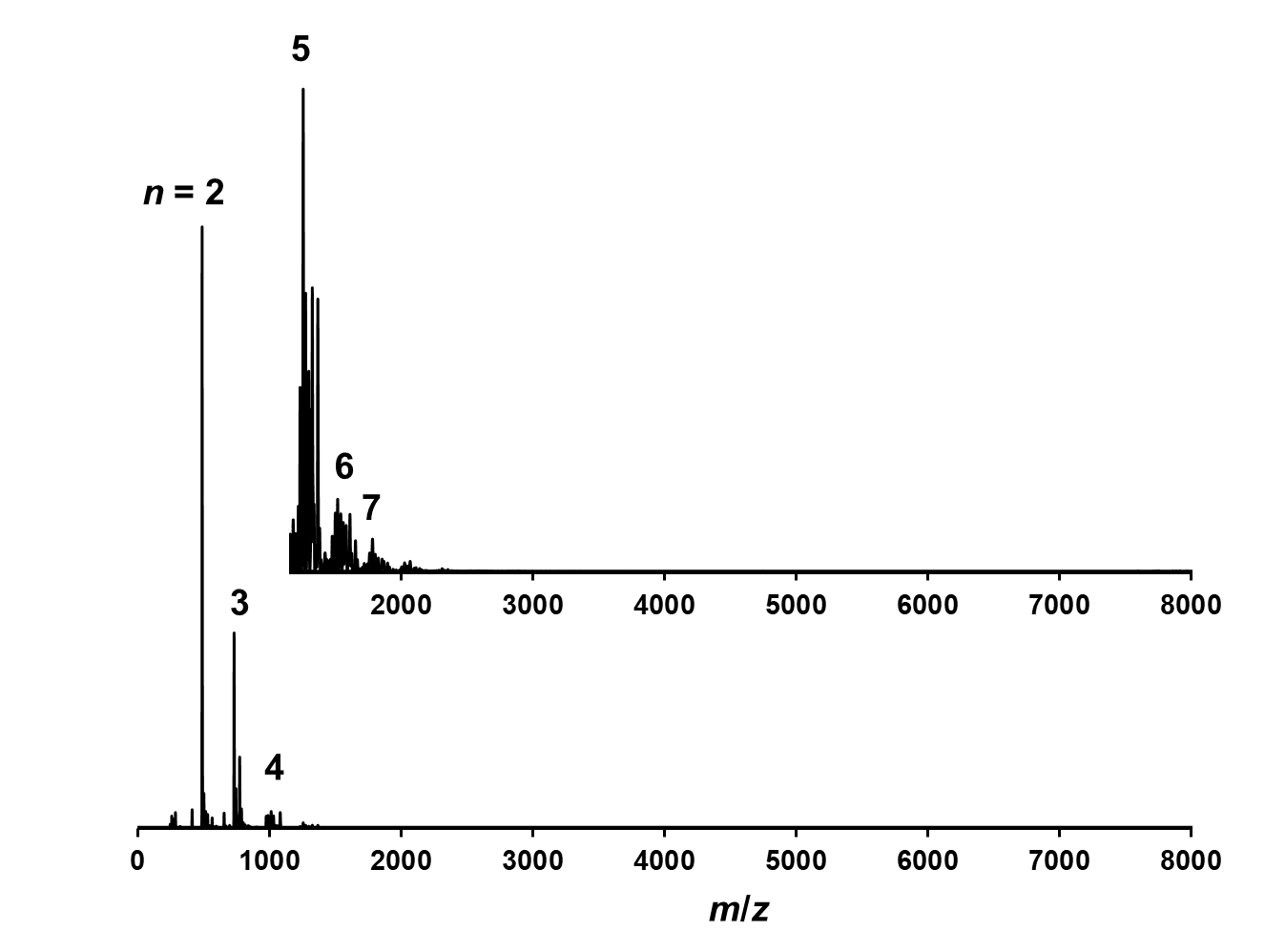


**Figure S4. MALDI** spectrum of **powder 1**. *n* refers to the number of triphenylamine units in the polymer; therefore, the difference between the main segment peaks is 245.3. The peaks among the main segment peaks indicate fragmentation between C-N bonds. ^1^


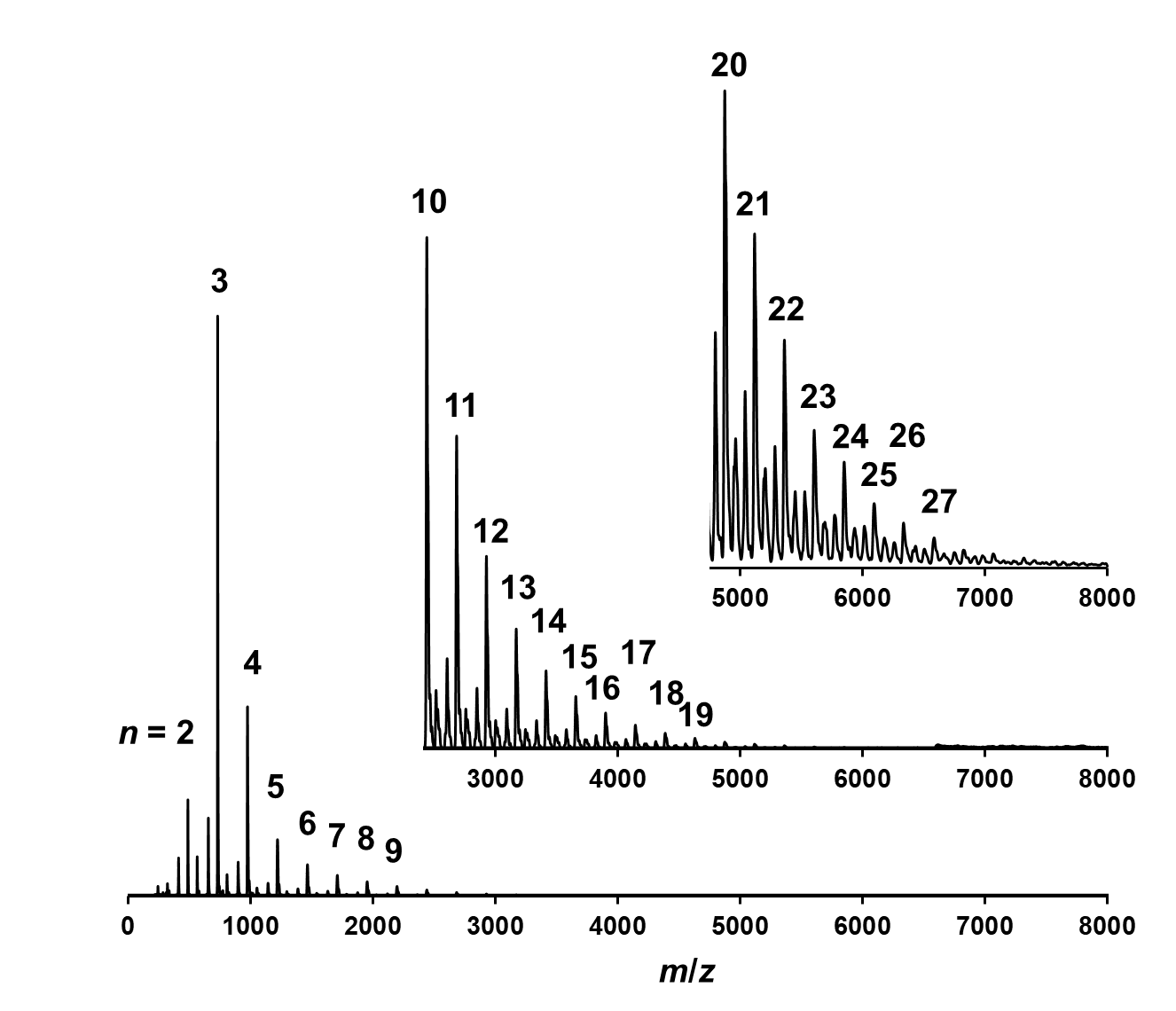


**Figure S5. MALDI** spectrum of **powder 3**. *n* refers to the number of triphenylamine units in the polymer; therefore, the difference between the main segment peaks is 245.3. The peaks among the main segment peaks indicate fragmentation between C-N bonds. ^1^


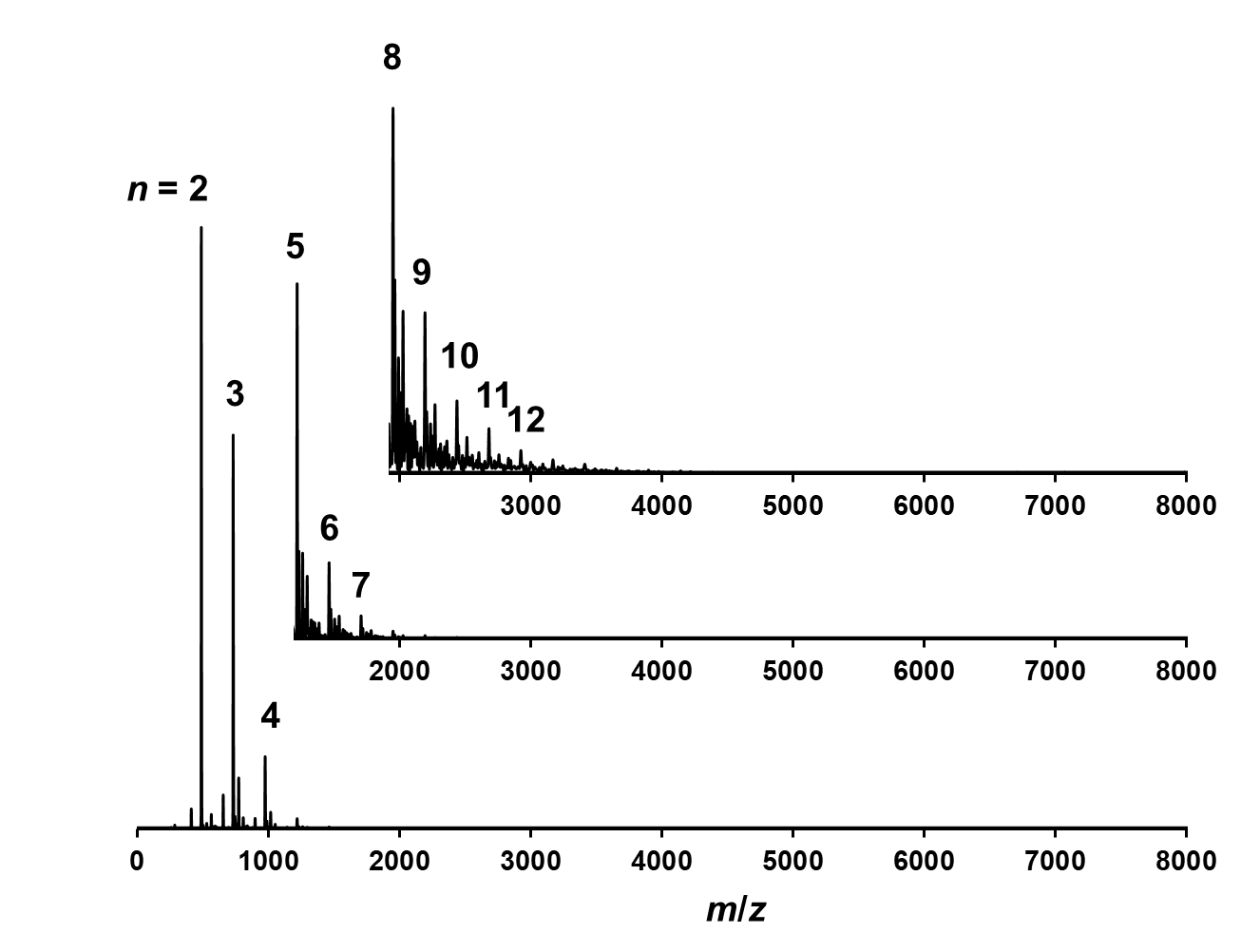


**Figure S6. MALDI** spectrum of **powder 4**. *n* refers to the number of triphenylamine units in the polymer; therefore, the difference between the main segment peaks is 245.3. The peaks among the main segment peaks indicate fragmentation between C-N bonds. ^1^


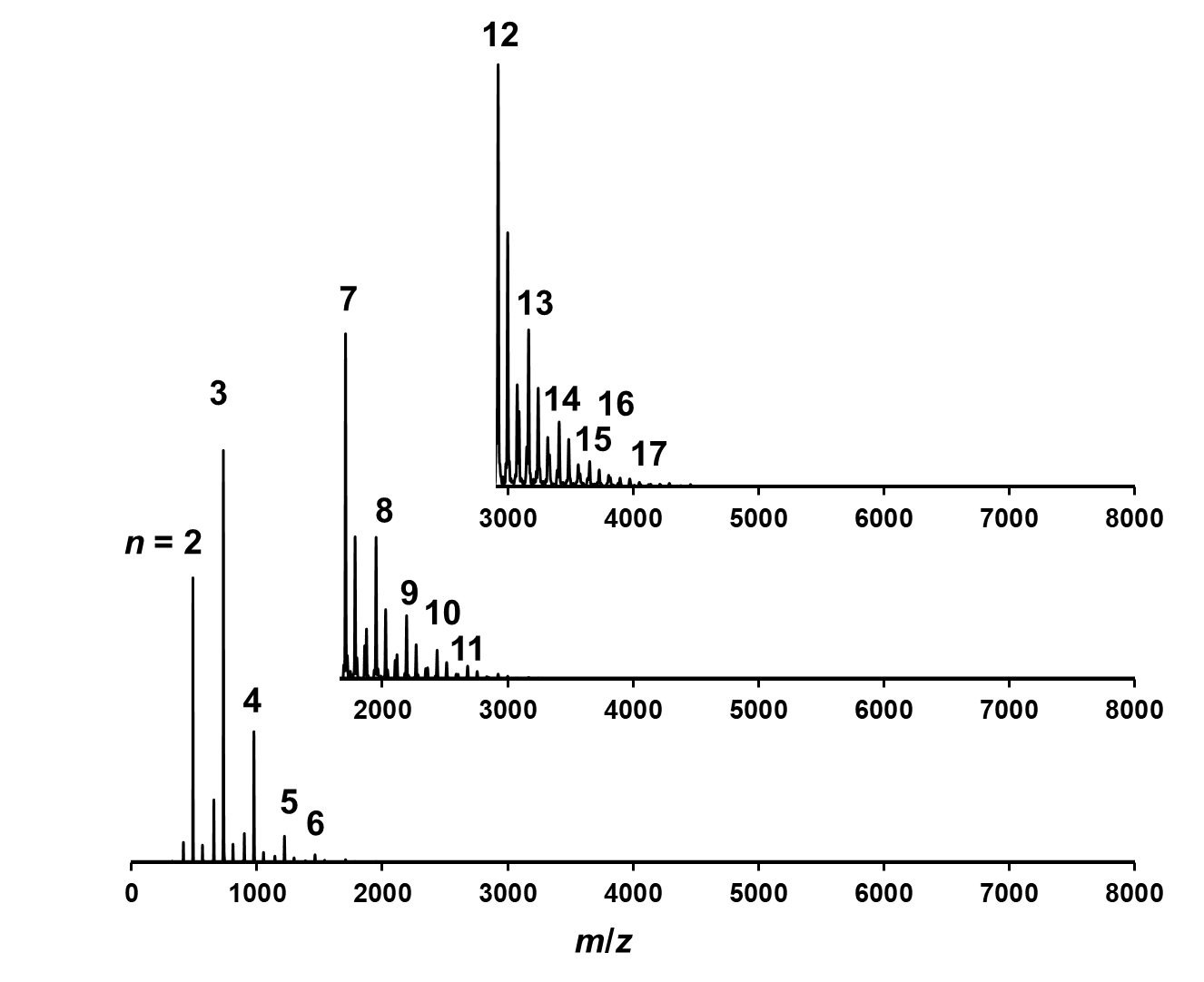


**Figure S7. MALDI** spectrum of **powder 5**. *n* refers to the number of triphenylamine units in the polymer; therefore, the difference between the main segment peaks is 245.3. The peaks among the main segment peaks indicate fragmentation between C-N bonds. ^1^


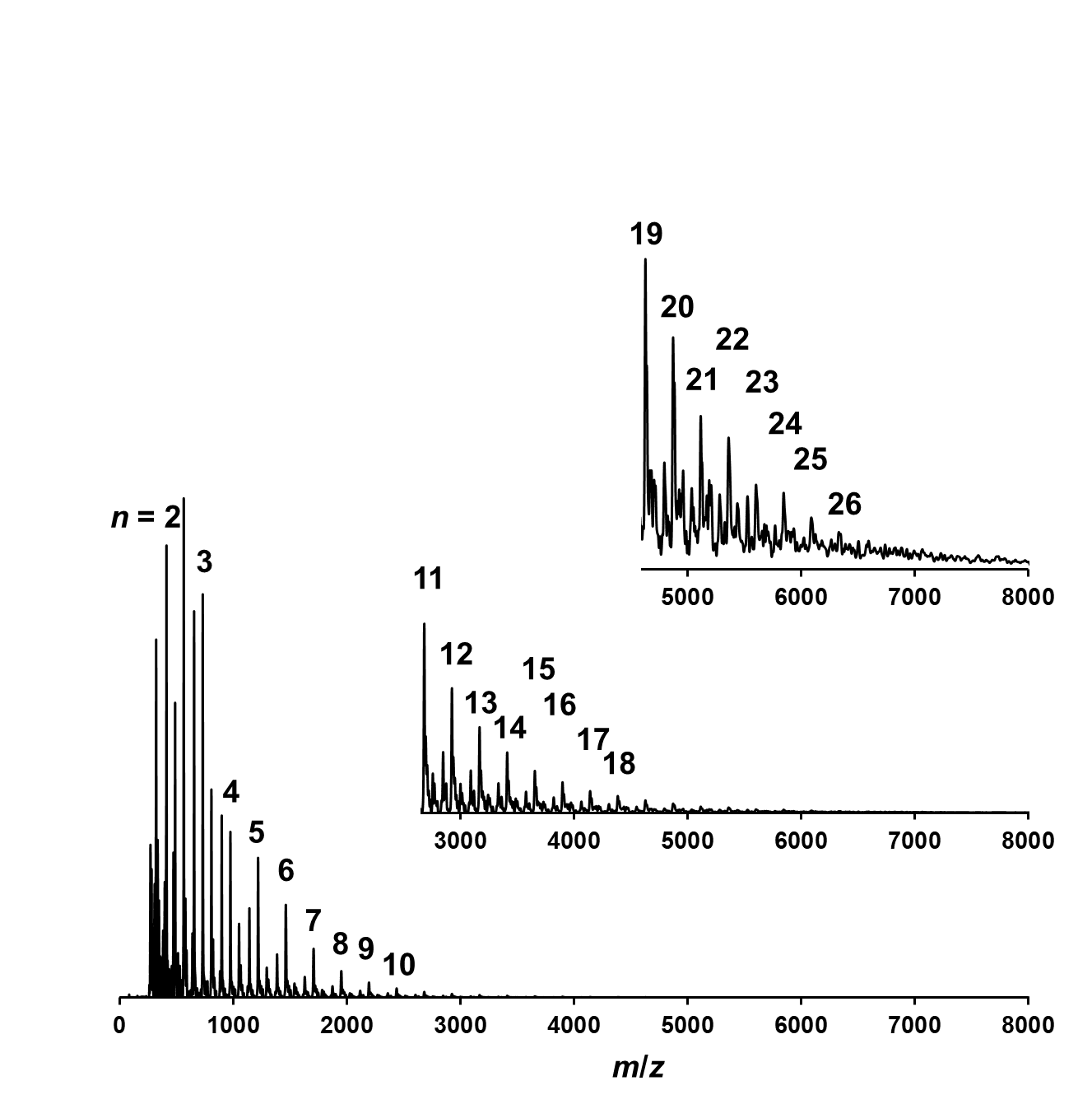


**Figure S8. MALDI** spectrum of **powder 6**. *n* refers to the number of triphenylamine units in the polymer; therefore, the difference between the main segment peaks is 245.3. The peaks among the main segment peaks indicate fragmentation between C-N bonds. ^1^


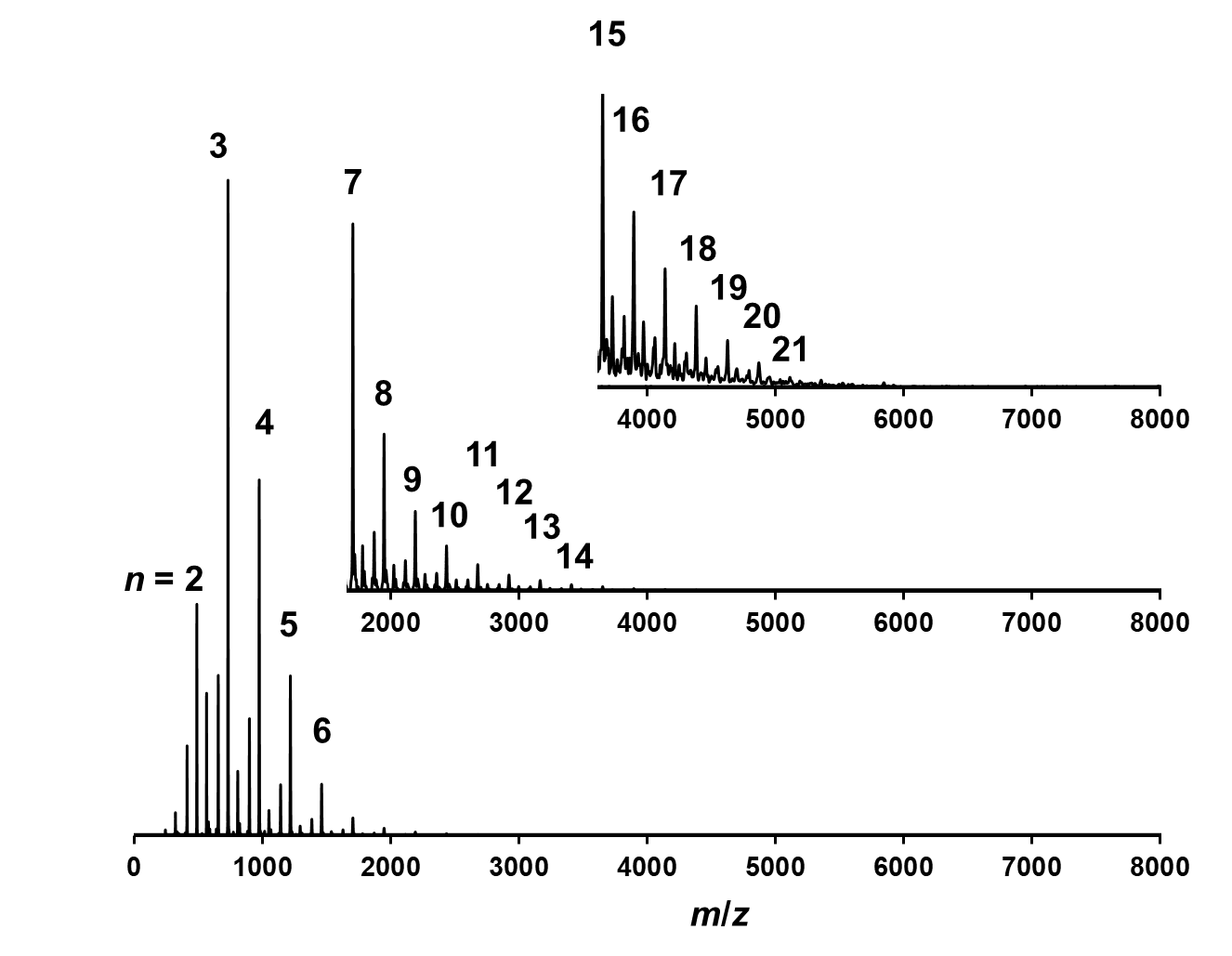


**Figure S9. MALDI** spectrum of **powder 7**. *n* refers to the number of triphenylamine units in the polymer; therefore, the difference between the main segment peaks is 245.3. The peaks among the main segment peaks indicate fragmentation between C-N bonds. ^1^


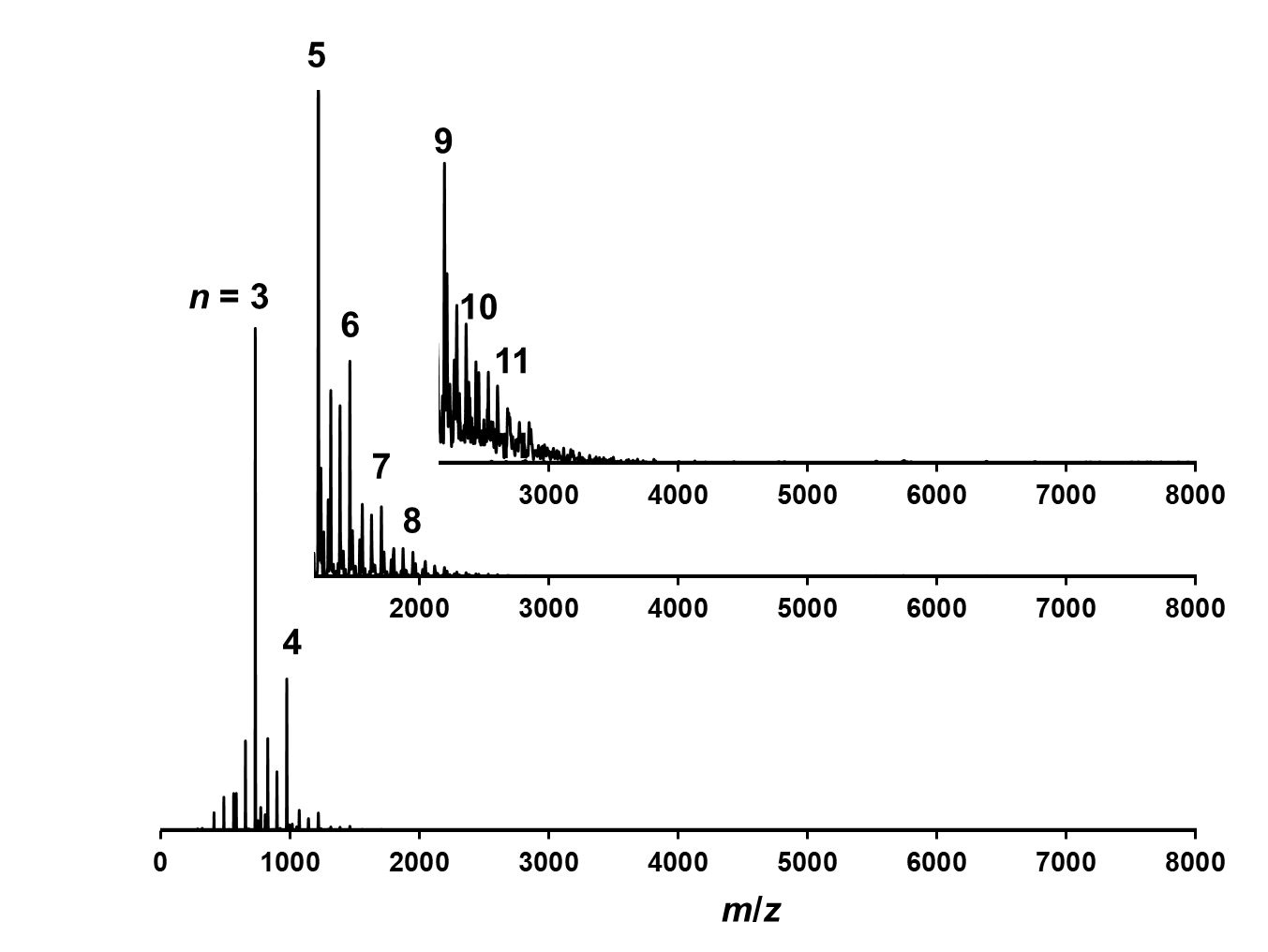


**Figure S10. MALDI** spectrum of **powder 8**. *n* refers to the number of triphenylamine units in the polymer; therefore, the difference between the main segment peaks is 245.3. The peaks among the main segment peaks indicate fragmentation between C-N bonds. ^1^


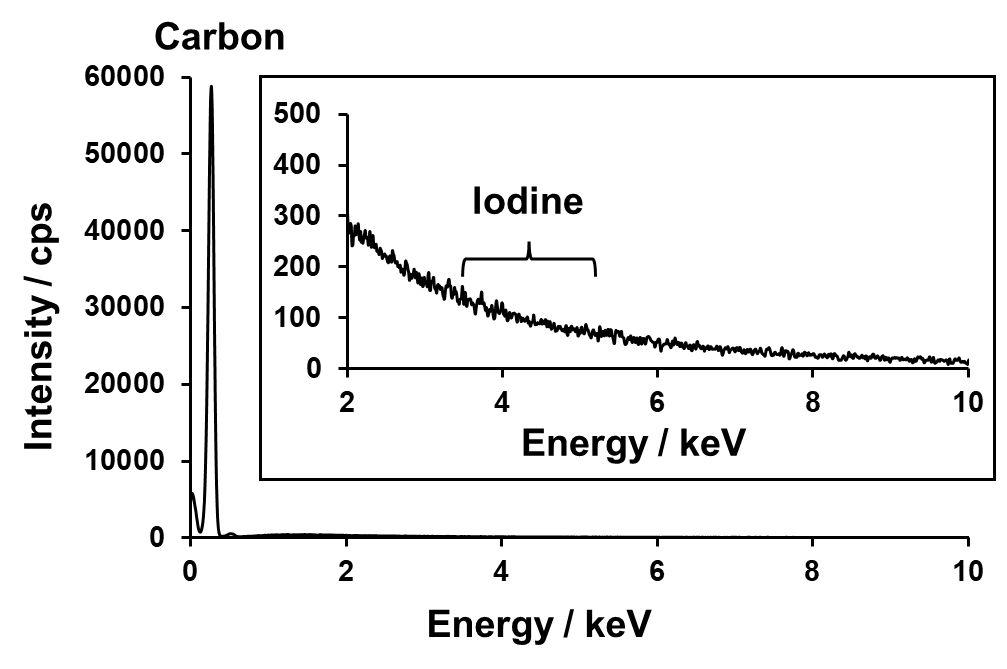


**Figure S11. SEM-EDX** spectrum of **powder 2**.


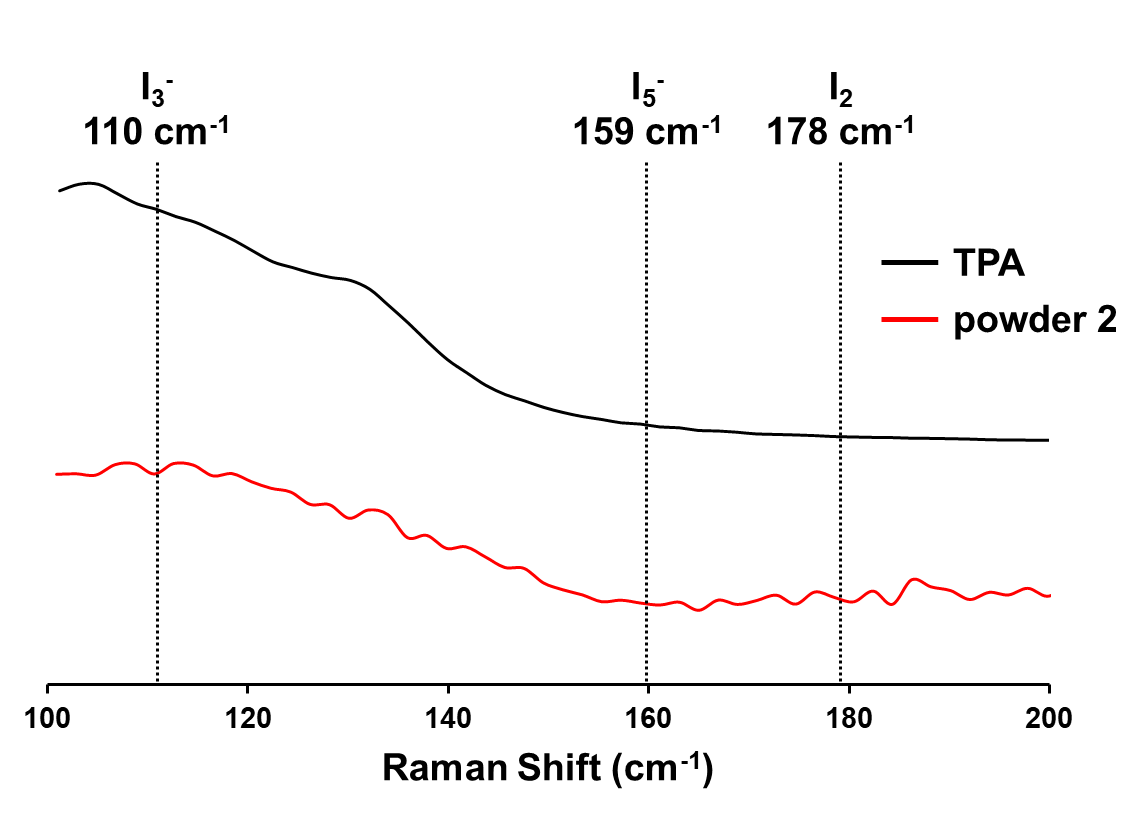


**Figure S12.** Raman spectra of **TPA** (black) and **powder 2** (red), in the range of 100–200 cm ^-1^.


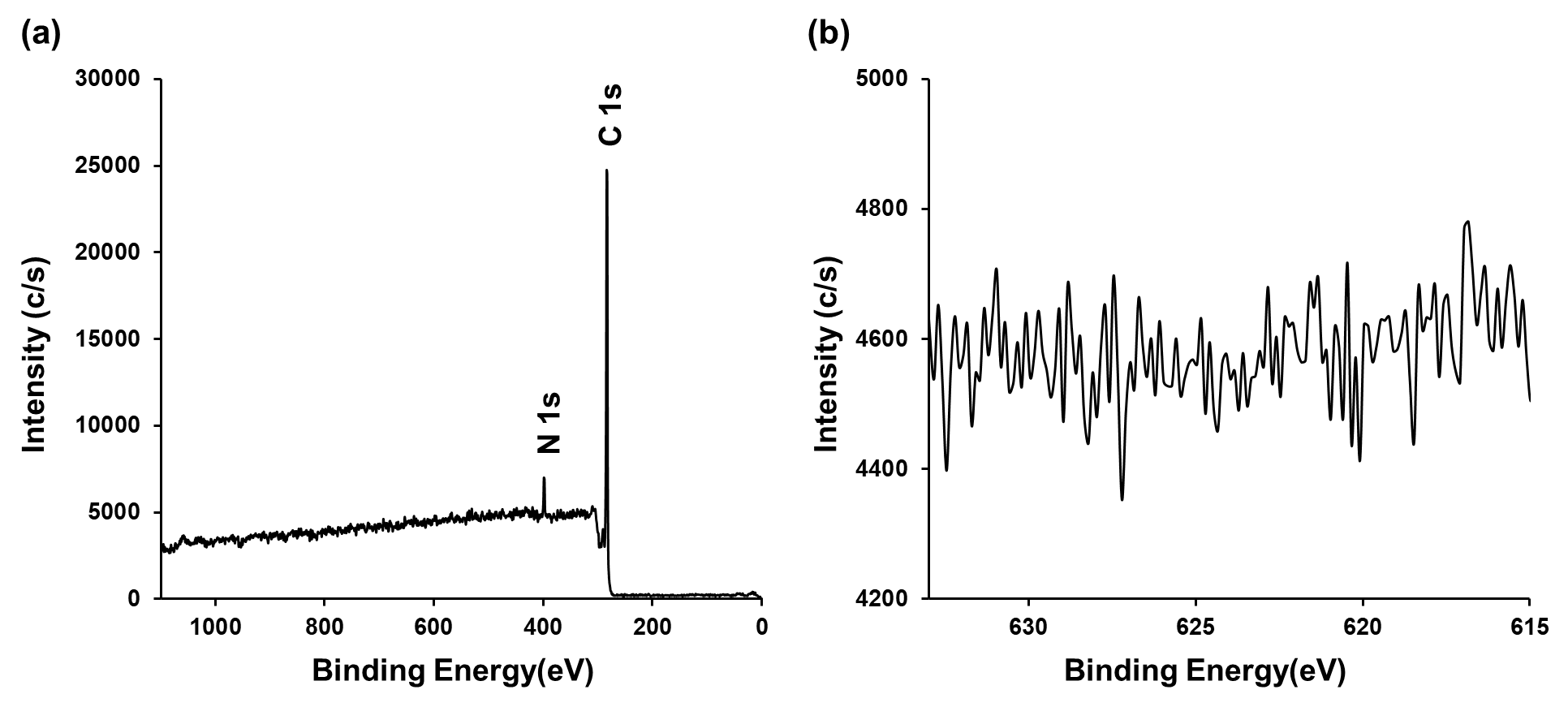


**Figure S13.** (a) Full-range **XPS** spectrum and (b) high resolution **XPS** spectrum of the I 3d peaks for **powder 2**.


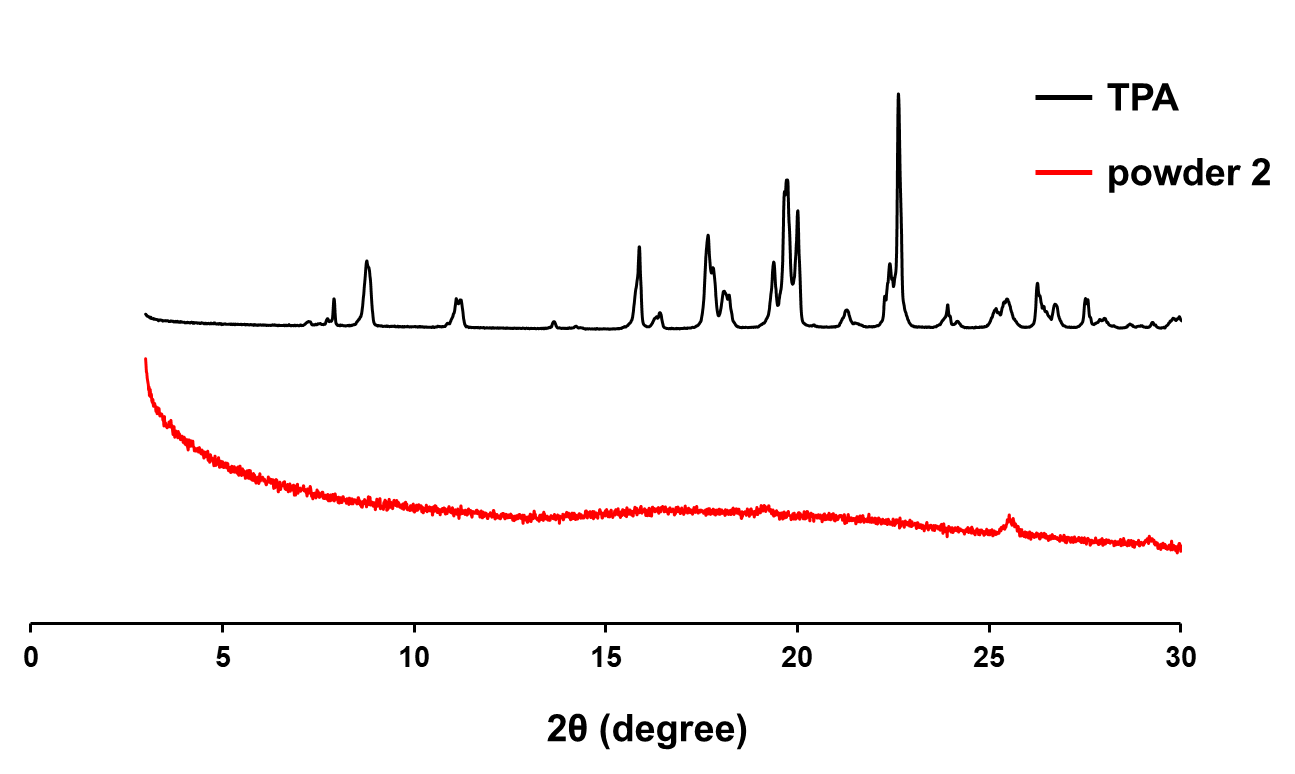


**Figure S14. XRD** patterns of **TPA** (black) and **powder 2** (red). A slight peak at about 25.6° (*d* = 0.349 nm) indicates the presence of intermolecular stacking, characteristic of conjugated aromatic molecules. ^2^


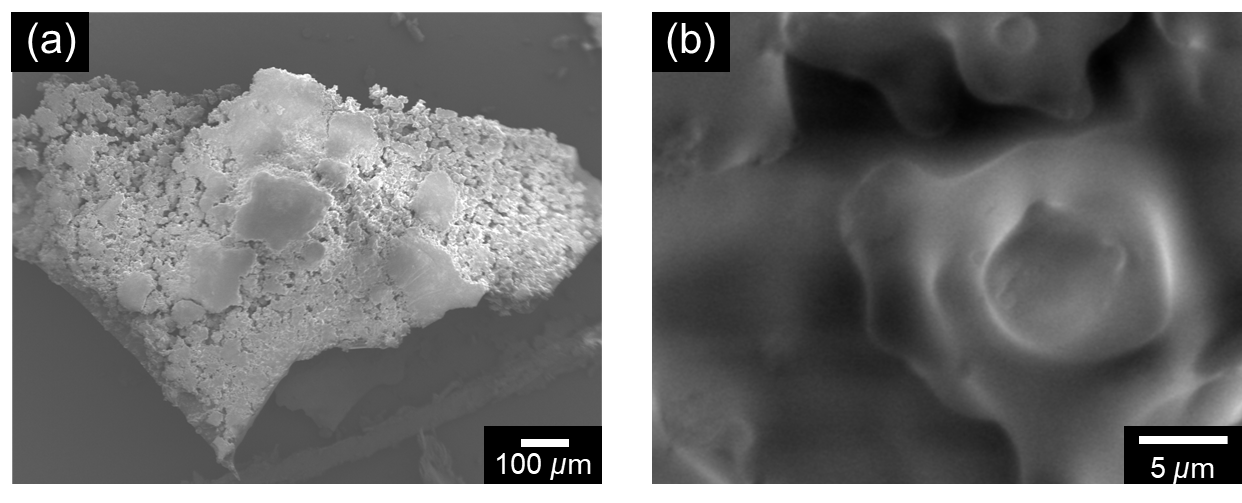


**Figure S15. SEM** images of (a) the shape of the particles and (b) the surface at high magnification of **powder 2**.


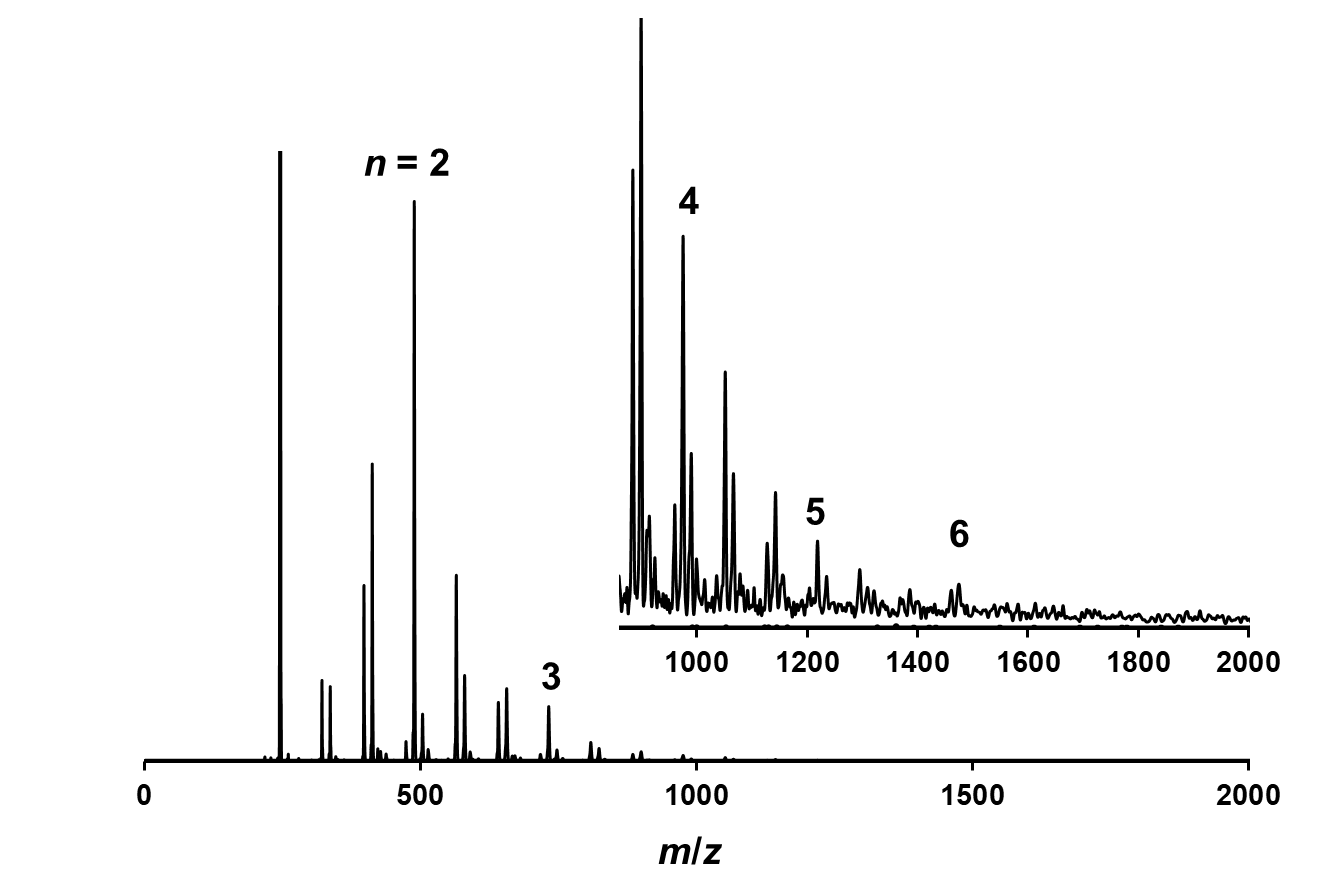


**Figure S16. MALDI** spectra of **pTPA** film. *n* refers to the number of triphenylamine units in the polymer; therefore, the difference between the main segment peaks is 245.3. The peaks among the main segment peaks indicate fragmentation between C-N bonds. ^1^


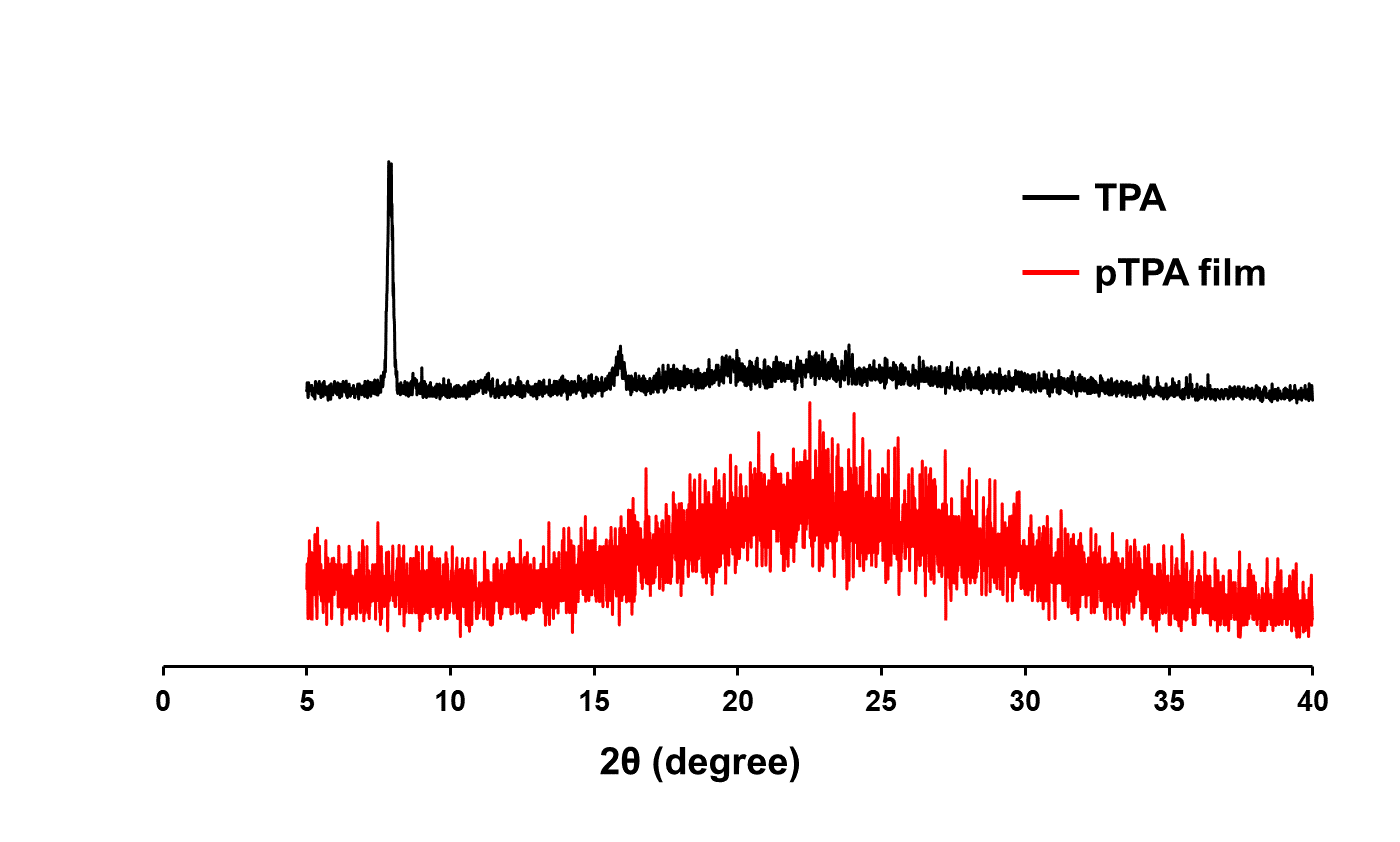


**Figure S17. XRD** patterns of **TPA** (black) and **pTPA** film (red).


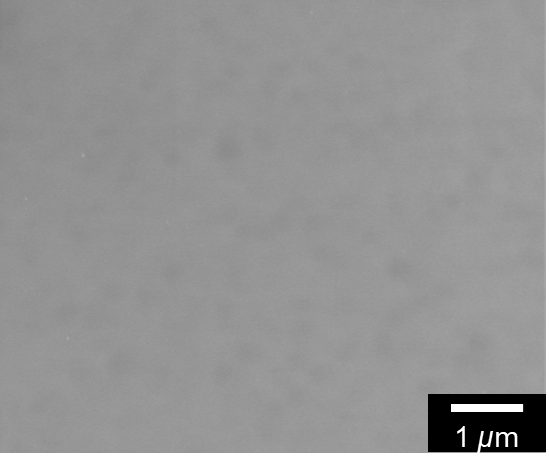


**Figure S18. SEM** image of **pTPA** film.


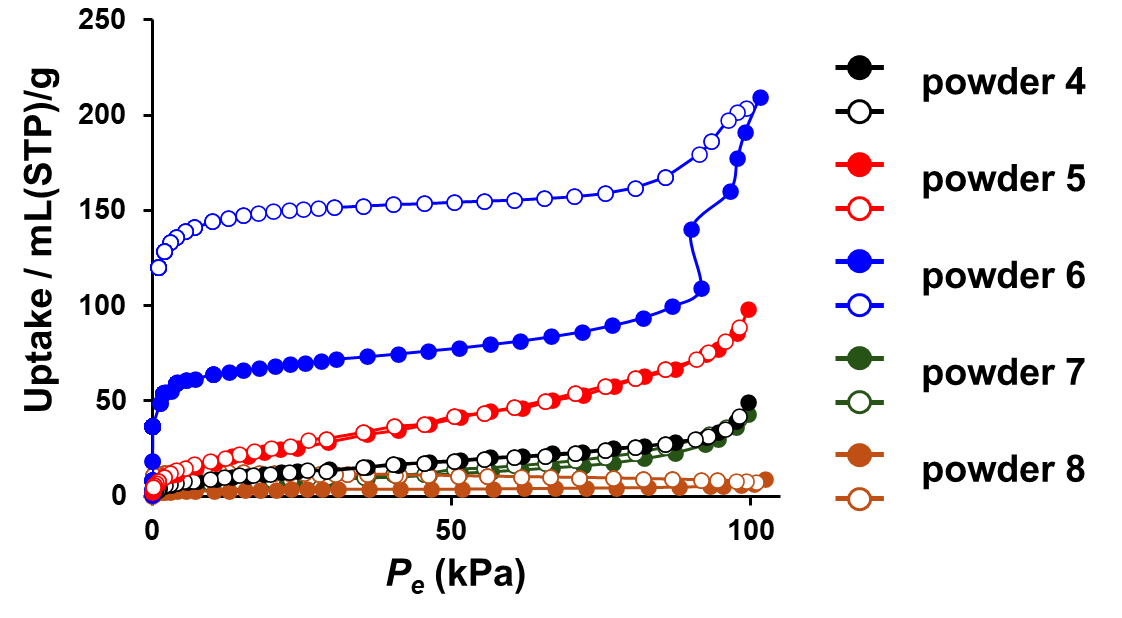


**Figure S19.** N_2_ adsorption isotherms of **powder 4** (black), **powder 5** (red), **powder 6** (blue), **powder 7** (green), and **powder 8** (brown), measured at 77 K. Solid symbols denote the adsorption isotherms and open symbols denote the desorption isotherms.


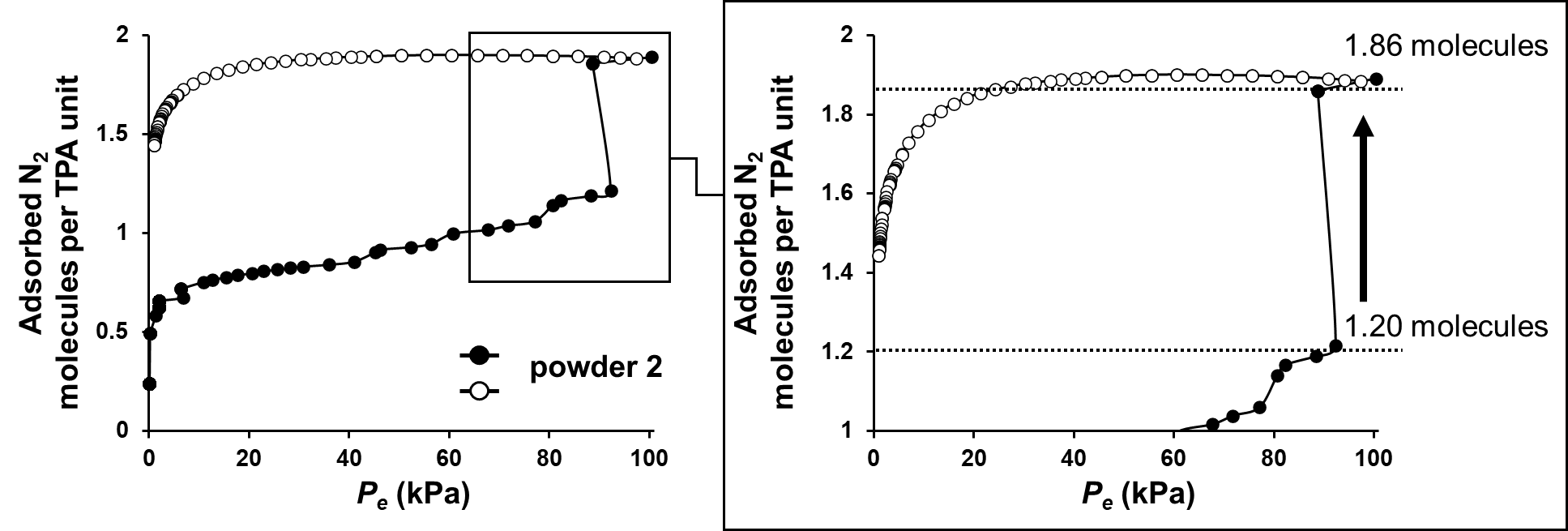


**Figure S20.** N_2_ adsorption per **TPA** unit of **powder 2** at 77 K. Solid symbols denote the adsorption isotherms and open symbols denote the desorption isotherms.


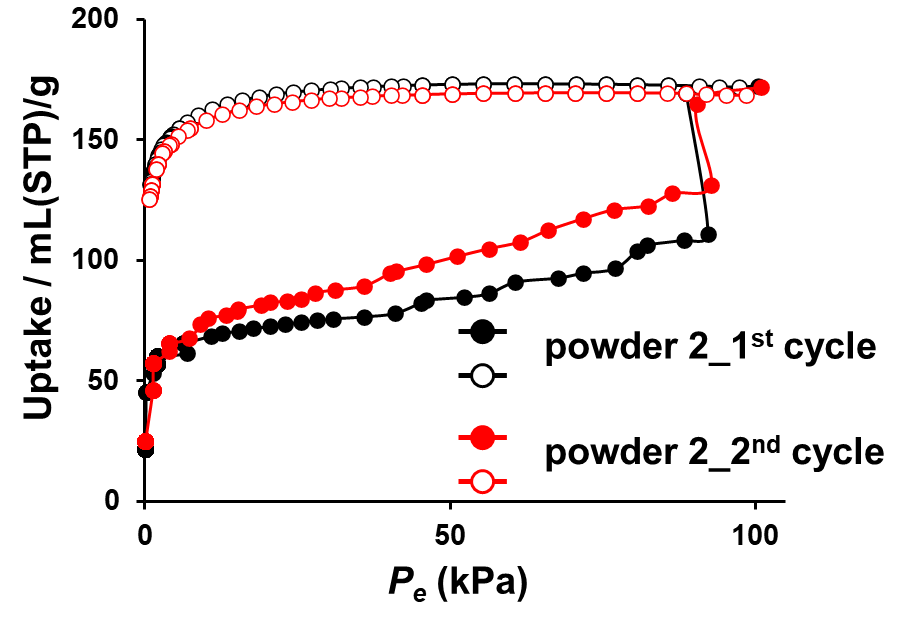


**Figure S21.** 1^st^ (black) and 2^nd^ (red) cycles of N_2_ adsorption isotherms for **powder 2**, measured at 77 K. Solid symbols denote the adsorption isotherms and open symbols denote the desorption isotherms.


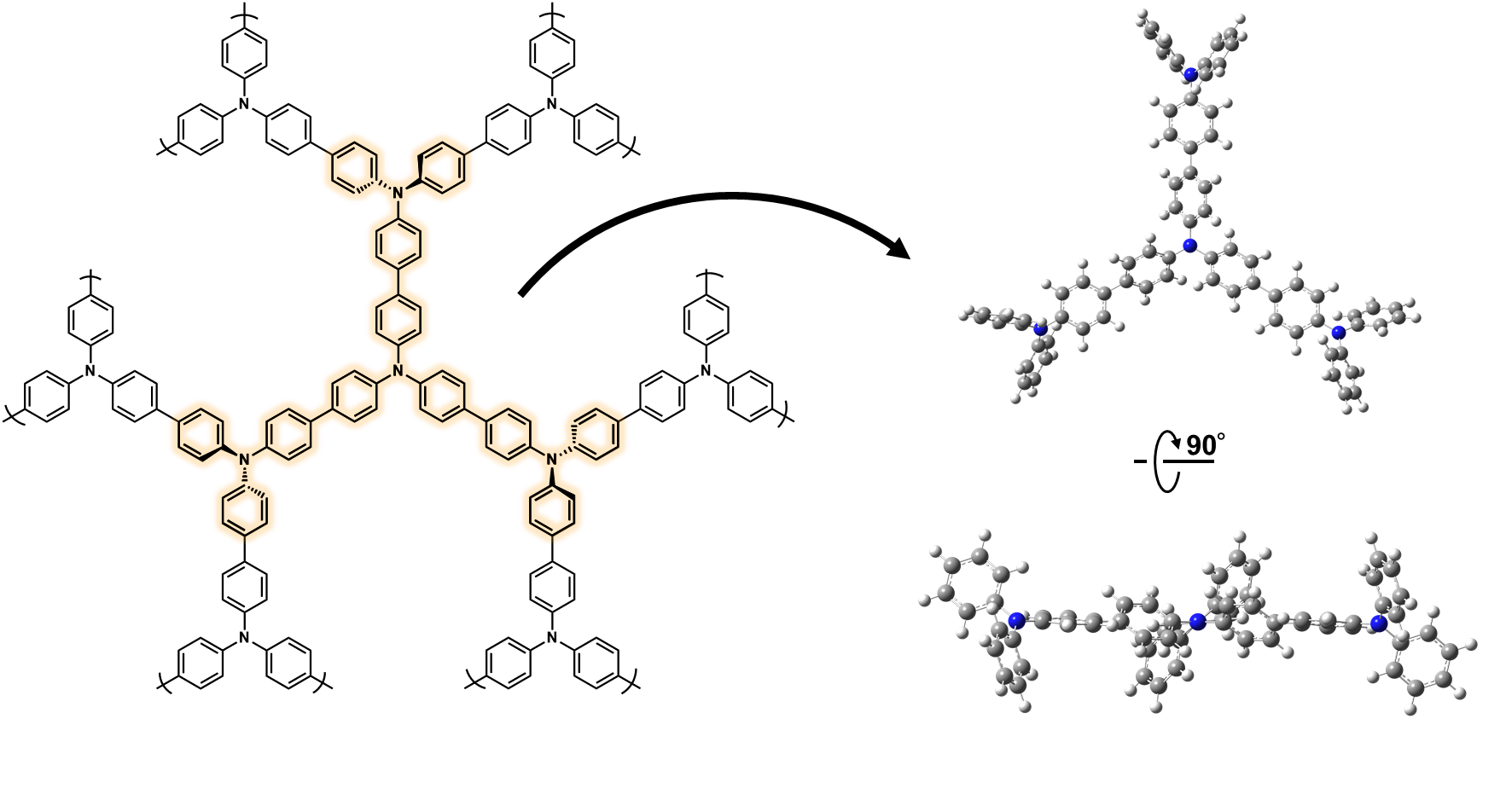


**Figure S22. DFT** (B3LYP/6-31G (d,p)) optimized structures of **pTPA** substructure.


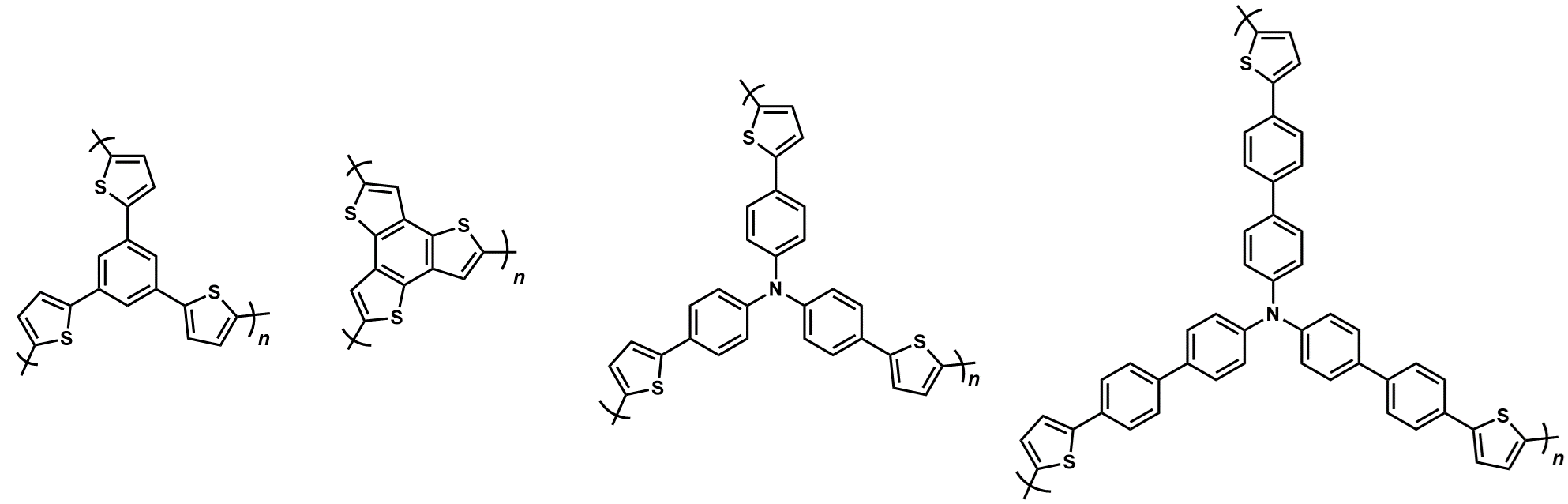


**Figure S23.** Some examples of **POP**s which can be synthesized via iodine-based chemical polymerization.

**Table S1.** Assignments of Raman spectra of **pTPA**.

| assignments | Raman shift (cm^-1^) |
| --- | --- |
| Ring stretching | 787 |
| Ring deformation | 818 |
| C-N stretching | 1170 |
| C-C stretching | 1289 |
| C-H in-plane bending | 1520 |
| C＝C stretching | 1610 |

**Table S2.** Absorbance of the peak at 747 cm^-1^ corresponding to the C-H bending vibration of monosubstituted benzene from the **IR** spectra of **TPA** and **powders 1–8**, percentage of remaining C-H bonds of monosubstituted benzene, and degree of polymerization. The degree of polymerization was 35% and 36% for **powder 6** (reaction time: 48 hours) and **powder 2** (reaction time: 20 hours), respectively, and therefore the reaction was considered to be complete in 20 hours. Therefore, in **Table 2**, the difference in the **BET** specific surface area between **powder 6** (252.80 m^2^/g) and **powder 2** ((2.7±0.2) ×10^2^ m^2^/g) was attributed to this slight difference in degree of polymerization and the dispersion of the measurement data (standard deviation).

| Sample | Absorbance of the peak at 747 cm^-1^  / a.u. | Percentage of remaining C-H bonds of monosubstituted benzene / % | | Degree of polymerization/ % | |  |
| --- | --- | --- | --- | --- | --- | --- |
| TPA | 1.503 | | 100 | | 0 | |
| powder 1 | 1.336 | | 89 | | 11 | |
| powder 2 | 0.965 | | 64 | | 36 | |
| powder 3 | 0.928 | | 61 | | 39 | |
| powder 4 | 1.195 | | 80 | | 20 | |
| powder 5 | 1.176 | | 78 | | 22 | |
| powder 6 | 0.972 | | 65 | | 35 | |
| powder 7 | 0.984 | | 66 | | 34 | |
| powder 8 | 1.173 | | 78 | | 22 | |

**Cartesian coordinates of optimized geometries**


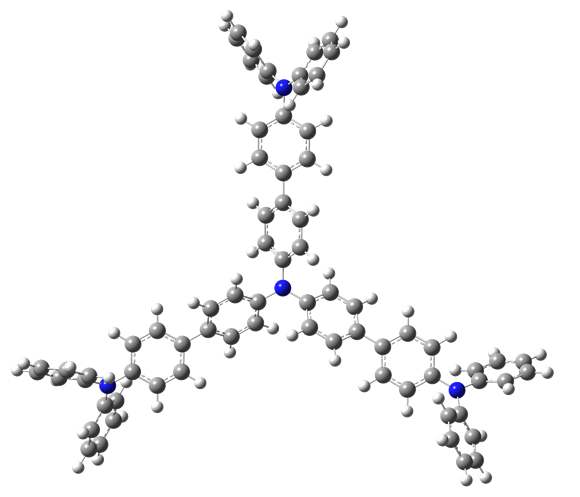


| atom | X | Y | Z |
| --- | --- | --- | --- |
| C | 2.195823 | -2.91491 | 0.791881 |
| C | 1.107099 | -2.05054 | 0.802867 |
| C | 1.107594 | -0.89149 | 0.010157 |
| C | 2.233991 | -0.62721 | -0.7854 |
| C | 3.322619 | -1.49158 | -0.7788 |
| C | 3.333321 | -2.65861 | 0.00566 |
| H | 2.179934 | -3.78637 | 1.439581 |
| H | 0.252845 | -2.26264 | 1.436955 |
| H | 2.245522 | 0.25421 | -1.41766 |
| H | 4.166175 | -1.27706 | -1.42806 |
| N | -0.00371 | -0.00753 | 0.012712 |
| C | -1.32506 | -0.52755 | 0.01271 |
| C | -2.32645 | 0.054876 | 0.806121 |
| C | -1.6622 | -1.63518 | -0.78168 |
| C | -3.62062 | -0.45254 | 0.796036 |
| H | -2.08061 | 0.900962 | 1.439001 |
| C | -2.95637 | -2.14273 | -0.77406 |
| H | -0.9061 | -2.08749 | -1.41456 |
| C | -3.97065 | -1.56501 | 0.010296 |
| H | -4.36613 | -0.00029 | 1.443235 |
| H | -3.19511 | -2.97996 | -1.4231 |
| C | 0.20639 | 1.396642 | 0.01114 |
| C | 1.213636 | 1.973788 | 0.801145 |
| C | -0.5879 | 2.241888 | -0.7803 |
| C | 1.419914 | 3.348446 | 0.790299 |
| H | 1.825876 | 1.33874 | 1.432553 |
| C | -0.38143 | 3.616594 | -0.77358 |
| H | -1.3599 | 1.812744 | -1.4101 |
| C | 0.628181 | 4.207166 | 0.007158 |
| H | 2.185752 | 3.768696 | 1.435339 |
| H | -0.99019 | 4.241279 | -1.42039 |
| C | 4.493647 | -3.57793 | 0.003768 |
| C | 5.808256 | -3.09761 | -0.13146 |
| C | 4.328433 | -4.96801 | 0.136861 |
| C | 6.902295 | -3.95504 | -0.12765 |
| H | 5.981445 | -2.02771 | -0.19914 |
| C | 5.414353 | -5.83586 | 0.127807 |
| H | 3.327101 | -5.38235 | 0.206946 |
| C | 6.722895 | -5.34227 | -0.00163 |
| H | 7.906041 | -3.55227 | -0.21104 |
| H | 5.253445 | -6.90549 | 0.209688 |
| C | 0.84881 | 5.671015 | 0.004511 |
| C | -0.2214 | 6.573672 | -0.12657 |
| C | 2.137946 | 6.217755 | 0.132987 |
| C | -0.0202 | 7.949143 | -0.12225 |
| H | -1.23624 | 6.1927 | -0.19137 |
| C | 2.3521 | 7.5912 | 0.124386 |
| H | 2.99509 | 5.554302 | 0.199122 |
| C | 1.273491 | 8.48204 | 0.000153 |
| H | -0.86825 | 8.620737 | -0.20266 |
| H | 3.360769 | 7.982491 | 0.202765 |
| C | -5.34929 | -2.10437 | 0.00799 |
| C | -5.59721 | -3.48183 | -0.12775 |
| C | -6.46647 | -1.26089 | 0.14064 |
| C | -6.88937 | -3.99435 | -0.12434 |
| H | -4.76048 | -4.17064 | -0.19604 |
| C | -7.7634 | -1.76108 | 0.131234 |
| H | -6.31953 | -0.18721 | 0.210399 |
| C | -7.99694 | -3.13999 | 0.002096 |
| H | -7.04771 | -5.06419 | -0.20856 |
| H | -8.60592 | -1.08269 | 0.212372 |
| N | 1.485259 | 9.885023 | -0.00228 |
| N | 7.835685 | -6.22247 | -0.00434 |
| N | -9.31816 | -3.65682 | -0.00182 |
| C | 0.745096 | 10.71609 | -0.88762 |
| C | 0.225926 | 11.94349 | -0.4452 |
| C | 0.52342 | 10.32328 | -2.21744 |
| C | -0.49006 | 12.75943 | -1.31864 |
| H | 0.388202 | 12.25068 | 0.582437 |
| C | -0.20958 | 11.138 | -3.07783 |
| H | 0.927762 | 9.379839 | -2.5684 |
| C | -0.71728 | 12.36195 | -2.63768 |
| H | -0.88452 | 13.70568 | -0.9593 |
| H | -0.37124 | 10.81847 | -4.10343 |
| H | -1.2818 | 12.99717 | -3.3132 |
| C | 2.439061 | 10.46356 | 0.879838 |
| C | 2.535094 | 10.02899 | 2.211702 |
| C | 3.298137 | 11.48009 | 0.432272 |
| C | 3.476878 | 10.59411 | 3.069021 |
| H | 1.869656 | 9.249253 | 2.566584 |
| C | 4.223899 | 12.05166 | 1.302664 |
| H | 3.233624 | 11.8167 | -0.59694 |
| C | 4.323893 | 11.6109 | 2.623728 |
| H | 3.537156 | 10.24589 | 4.096261 |
| H | 4.880627 | 12.83705 | 0.939374 |
| H | 5.051473 | 12.05371 | 3.296817 |
| C | -9.66815 | -4.71063 | -0.8904 |
| C | -10.4701 | -5.77608 | -0.45079 |
| C | -9.21847 | -4.701 | -2.2206 |
| C | -10.8192 | -6.80108 | -1.32763 |
| H | -10.8161 | -5.79301 | 0.577217 |
| C | -9.55765 | -5.74027 | -3.08442 |
| H | -8.60442 | -3.87736 | -2.56894 |
| C | -10.3628 | -6.79392 | -2.64717 |
| H | -11.4405 | -7.61756 | -0.97062 |
| H | -9.20119 | -5.71661 | -4.11033 |
| H | -10.6309 | -7.59813 | -3.3253 |
| C | -10.296 | -3.12243 | 0.881778 |
| C | -9.96714 | -2.82456 | 2.214086 |
| C | -11.6063 | -2.88652 | 0.435463 |
| C | -10.9271 | -2.2935 | 3.072996 |
| H | -8.95889 | -3.01123 | 2.568066 |
| C | -12.5638 | -2.37247 | 1.30742 |
| H | -11.8663 | -3.10894 | -0.59395 |
| C | -12.2315 | -2.06781 | 2.628851 |
| H | -10.6551 | -2.0691 | 4.100507 |
| H | -13.5726 | -2.19611 | 0.945067 |
| H | -12.9785 | -1.66058 | 3.303131 |
| C | 8.922421 | -5.99505 | -0.89292 |
| C | 10.24679 | -6.1526 | -0.45351 |
| C | 8.688253 | -5.61128 | -2.22328 |
| C | 11.30829 | -5.93845 | -1.33024 |
| H | 10.43578 | -6.4433 | 0.574373 |
| C | 9.75717 | -5.38156 | -3.08695 |
| H | 7.667682 | -5.49497 | -2.57203 |
| C | 11.07281 | -5.54732 | -2.64971 |
| H | 12.32642 | -6.06487 | -0.97313 |
| H | 9.557419 | -5.08537 | -4.11286 |
| H | 11.9028 | -5.3744 | -3.32774 |
| C | 7.867211 | -7.33392 | 0.882437 |
| C | 7.445555 | -7.19558 | 2.214655 |
| C | 8.32237 | -8.58615 | 0.439269 |
| C | 7.470961 | -8.28977 | 3.076879 |
| H | 7.099656 | -6.22927 | 2.565853 |
| C | 8.361452 | -9.66958 | 1.314473 |
| H | 8.644286 | -8.70166 | -0.59018 |
| C | 7.932389 | -9.53166 | 2.635986 |
| H | 7.141196 | -8.16473 | 4.104363 |
| H | 8.716591 | -10.6311 | 0.954685 |
| H | 7.957427 | -10.38 | 3.312861 |

**Reference**

(1) Sprick, R. S.; Hoyos, M.; Morrison, J. J.; Grace, I. M.; Lambert, C.; Navarro, O.; Turner, M. L. Triarylamine Polymers of Bridged Phenylenes by (N-Heterocyclic Carbene)-Palladium Catalysed C–N Coupling. *Journal of Materials Chemistry C* **2013**, *1* (20). DOI: 10.1039/c3tc30368c

(2) Zhang, Y.; Liu, J.; Wu, G.; Chen, W. Porous Graphitic Carbon Nitride Synthesized Via Direct Polymerization of Urea for Efficient Sunlight-Driven Photocatalytic Hydrogen Production. *Nanoscale* **2012**, *4* (17), 5300-5303. DOI: 10.1039/c2nr30948c
